# Psychological Therapy Outcomes and Engagement in People of Different Religions

**DOI:** 10.1001/jamanetworkopen.2025.4026

**Published:** 2025-04-08

**Authors:** Zainab Shafan-Azhar, Jae Won Suh, Henry Delamain, Laura-Louise Arundell, Syed Ali Naqvi, Tania Knight, Sarah Ellard, Stephen Pilling, Rob Saunders, Joshua E. J. Buckman

**Affiliations:** 1 CORE Data Lab, Centre for Outcomes Research and Effectiveness, Research Department of Clinical, Educational and Health Psychology, University College London, London, United Kingdom; 2 National Collaborating Centre for Mental Health, Royal College of Psychiatrists, London, United Kingdom; 3 North East London National Health Service Foundation Trust, London, United Kingdom; 4 Whittington Health National Health Service Trust, London, United Kingdom; 5 North London National Health Service Foundation Trust, London, United Kingdom; 6 Camden and Islington National Health Service Talking Therapies for Anxiety and Depression Services, North London National Health Service Foundation Trust, London, United Kingdom; 7 Children and Young People’s Mental Health Coalition, United Kingdom

## Abstract

**Importance:**

Identifying whether people of minoritized religious identities are less likely to benefit from psychological therapy is key to tackling inequalities in mental health treatment.

**Objective:**

To assess inequalities in the effectiveness of routinely delivered psychological therapy across religious groups and by the intersections with ethnicity.

**Design, Setting, and Participants:**

Retrospective cohort study including all patients who completed a course of treatment at 5 London-based National Health Service Talking Therapies for anxiety and depression (NHS TTad) services between 2011 and 2020. Individuals reported their religion using routine patient records collected by the services. Data were analyzed from September 2023 to October 2024.

**Exposures:**

Self-identified religion was categorized into (1) no religion, (2) Christian, (3) Muslim, and (4) other (which was further categorized into Buddhist, Hindu, Jewish, Sikh, and any other in a sensitivity analysis). Ethnicity was conceptualized as a potential confounder and separately as an effect modifier. Self-reported ethnicity was categorized based on UK Census codes into Asian, Black, mixed race, White, and other ethnic groups.

**Main Outcomes and Measures:**

Psychological treatment outcomes used to assess NHS TTad services nationally, including reliable recovery, recovery, and reliable deterioration. Dropout from treatment was also examined. These outcomes were defined based on pre-post treatment changes in depression and anxiety symptom measures according to national guidelines.

**Results:**

A total of 70 098 patients with data on self-reported religion were included in the study (mean [SD] age at referral, 39.2 [14.1] years; 47 797 [68.2%] female). After adjusting for sociodemographic, treatment-related, and clinical characteristics, the odds of reliable recovery were higher in patients who did not have any religious belief (odds ratio [OR], 1.34; 95% CI, 1.26-1.42) or self-reported Christian (OR, 1.39; 95% CI, 1.31-1.48) and other religion (OR, 1.25; 95% CI, 1.17-1.34) compared with Muslim patients. While treatment outcomes improved each year in all groups, Muslim patients remained least likely to improve and more likely to deteriorate. There were interactions between religion and ethnicity; in particular, Muslim patients of White or other ethnic backgrounds had worse outcomes than Muslim patients of Asian, Black, or mixed race ethnic backgrounds and compared with non-Muslim patients of those ethnicities.

**Conclusions and Relevance:**

In England, patients who identified as Muslim, and particularly those of White or other ethnicities, had poorer outcomes from psychological therapies for depression and anxiety disorders than patients who reported no religion or any other religion. This may be partly due to unmeasured characteristics that warrant further investigation (eg, nationality and asylum-seeking or refugee status). Best practice guidelines on working with people of minoritized ethnicities may inform some of the changes needed to reduce inequalities, but must address religious identity separate from ethnicity, as well as their intersections.

## Key Points

**Question:**

Are there inequalities in psychological therapy outcomes by self-identified religion, and if so, what are the contributing factors?

**Findings:**

In this cohort study of 70 098 individuals in England, UK, Muslim patients were less likely to recover following psychological therapy than patients of all other religions or none after adjusting for sociodemographic, treatment-related, and clinical characteristics. Muslim patients of White or other ethnicities had worse outcomes than Asian, Black, and mixed race Muslim patients and patients of those ethnicities with other religious identities.

**Meaning:**

These findings suggest cultural adaptations at the organizational, therapist, and therapy levels should be considered to reduce inequalities in psychological therapy outcomes, particularly for Muslim patients of White or other ethnic backgrounds.

## Teaser

This cohort study evaluates whether a patient’s religion is associated with their outcomes in psychological therapy.

## Introduction

Religious identity can play an important role in the risk of and recovery from mental health problems.^1,2^ While many religions hold majority status globally or in other regions, they may be minoritized in specific local contexts, meaning they exist in smaller numbers and may have reduced social, cultural, or political visibility. People from minoritized religious groups may experience discrimination, harassment, and related mental distress, with potential compounded effects due to the intersectionality of religious and ethnic identities.^3,4,5^ Many countries have conducted research and implemented policies to promote culturally adapted psychological therapies for race or ethnicity,^6,7,8^ but there is a lack of similar research and policy to address inequalities related to religious identity.

In the United Kingdom (UK), religious identity is recognized as an important part of social identity^9,10,11^ and, like ethnicity, is a protected characteristic by law (UK Equality Act, 2010).^12^ All health bodies in the UK are required to collect and report data on patients’ protected characteristics to demonstrate equitable practices. While clinical guidance exists to improve care for patients of Black, Asian, and other minority ethnic identities,^13^ no similar best practice guides exist to address inequalities faced by people from minoritized religious groups.

To date, exploratory analyses of service-level (rather than patient-level) data from psychological therapies services in England have found preliminary evidence that Muslim patients may have worse outcomes following psychological therapy than patients of any other religion.^14,15^ However, both studies had high levels of missing data on religion (45%-60%) and only reported crude associations, limiting the ability to consider the independent association between religion and treatment outcomes. Furthermore, neither study was able to investigate intersectional effects with ethnicity, which could be crucial for identifying patient subgroups that face the greatest inequalities and for highlighting key areas where clinicians and referrers can focus their efforts; it could also reveal gaps in routine data collection that need to be filled to better understand these inequalities.^8,16,17^ Therefore, the aims of this study were to (1) investigate any inequalities in psychological treatment outcomes in patients of different religions (including those with no religion); (2) consider how outcomes for patients in each group have changed over time; and (3) examine potential intersectional effects between self-identified religion and ethnicity.

## Methods

### Data Source and Participants

Data from National Health Service (NHS) Talking Therapies for anxiety and depression (TTad) services in the North and Central East London NHS TTad Service Improvement and Research Network were used.^18^ NHS TTad services provide evidence-based psychological treatments for depression and anxiety disorders to adults in England, UK, using a stepped care model.^19,20^ Low-intensity therapies (eg, guided self-help) are typically provided for less severe presentations and high-intensity therapies (eg, cognitive behavioral therapy or counseling for depression) for more severe presentations or for diagnoses for which there is no evidence-based low-intensity therapy (eg, posttraumatic stress disorder or social phobia). A standardized dataset is collected at assessment across services, and measures of anxiety and depression symptoms are collected at each clinical contact, as per national mandates (see eTable 2 in Supplement 1). Self-identified religion is not a mandatory data collection item nationally.

For this study, data were drawn from Barnet, Enfield, Haringey, Barking and Dagenham, and Redbridge services owing to the mandated collection of self-reported religion in these services. The services were located in areas in London with high levels of diversity in religious and ethnic identities (see eTable 1 in Supplement 1 for the 2021 UK Census data). For example, 31% of census respondents self-identified as Muslim in the local authority of Redbridge, compared with 15% in London and 6.7% in England. A retrospective cohort was formed of all patients referred to these services between 2011 and 2020 (see eFigure in Supplement 1 for study flowchart). Only those who had undergone 2 or more treatment sessions and those who had been discharged were included, in line with national reporting methods.^21^ Reporting of religion was 93.4% complete in this cohort, and individuals whose religion was unknown were excluded from analyses.

NHS ethical approval was not required for this study (confirmed by the Health Research Authority). The data were provided by the NHS TTad services for evaluation as part of a wider service improvement project conducted in accordance with the procedures of the host institution and the NHS Trusts which operate the services. All participants consented for their deidentified data to be used as part of audits and service evaluations to inform service improvement. This study followed Strengthening the Reporting of Observational Studies in Epidemiology (STROBE) reporting guidelines.

### Outcomes

Treatment outcomes are based on performance metrics used by NHS TTad services nationally.^21^ The primary outcome was reliable recovery; if both criteria for reliable improvement and for recovery (as defined in the subsequent list) were met, then the patient was considered to have reliable recovery.

Other outcomes were defined as secondary. The first of these, recovery, was defined as having moved from caseness before treatment on either the depression measure (Patient Health Questionnaire 9 [PHQ-9]), the relevant anxiety measure (Generalized Anxiety Disorder 7 [GAD-7] or anxiety disorder specific measure [ADSM]), or both, to below caseness on both measures after treatment. Caseness refers to symptom thresholds likely to be sufficient to meet the diagnostic criteria for the disorder (≥10 on PHQ-9 or ≥8 on GAD-7) (see eTable 3 in Supplement 1 for ADSM thresholds).

The second outcome, reliable improvement, was defined as a patient’s PHQ-9 score, GAD-7 score (or other ADSM), or both, reducing by a magnitude above the error of measurement (≥6 points on PHQ-9 or ≥4 points on GAD-7) (see eTable 3 in Supplement 1 for ADSM thresholds) without meeting criteria for reliable deterioration on either measure. Reliable deterioration, the third secondary outcome, was defined as a patient’s scores on either PHQ-9, GAD-7 or ADSM, or both, increasing pre-post treatment by a magnitude above the threshold for the error of measurement.

Finally, dropout was defined as terminating treatment earlier than the clinician planned. All treating clinicians are trained on how to record the reason for ending treatment, as without this code a patient cannot be discharged. This data item is never missing for anyone who is discharged, and is only missing when a patient is still in treatment. When the episode of care is ended by the patient before the planned ending, this is recorded as the patient having dropped out of treatment.

### Exposure and Covariates

Self-reported religion was captured using a list of 158 faiths and denominations, aggregated into 14 categories within the NHS TTad dataset (including no religion, declined to disclose, and unknown). For this study, we categorized religion into the most prevalent higher-order categories in the services from which data were collected: (1) no religion, (2) Christian, (3) Muslim, and (4) other (Buddhist, Hindu, Jewish, Sikh, or any other). Based on existing evidence, we hypothesized that Muslim patients were likely to have poorer therapy outcomes and therefore used Muslim as the reference group. See eTable 4 in Supplement 1 for comparisons using other reference groups.

Ethnicity was conceptualized as a potential confounder and separately as an effect modifier. Patients self-reported their ethnicity using 2 linked lists of options. The first included higher-order categories used in UK Census,^22^ Asian, Black, mixed race, White, and other. The second list included subcategories of each higher-order category (see eTable 2 in Supplement 1). A range of clinical and demographic covariates were adjusted for as potential confounders, using definitions and categories as they appear in eTable 2 in Supplement 1.

### Statistical Analysis

Initially, summary statistics of the exposures and covariates previously mentioned were reported across categories of religion. Missing data were then imputed using multiple imputations with chained equations.^23^ Imputation models included all variables listed in eTable 2 in Supplement 1 and produced 50 imputed datasets; all variables had less than 50% missingness (Table 1). Sensitivity analyses using complete cases were also conducted.

**Table 1.** Participant Characteristics^a^

Characteristic	Participants, No. (%)			
	Muslim (n = 10 350)	No religion (n = 27 126)	Christian (n = 24 217)	Other religion (n = 8405)
Age at referral, mean (SD), y	37.7 (12.0)	35.9 (12.9)	42.3 (14.7)	42.4 (15.5)
Race and ethnicity				
White	3280 (31.7)	21 337 (78.7)	15 920 (65.7)	3909 (46.5)
Asian	4876 (47.1)	1251 (4.6)	594 (2.5)	3414 (40.6)
Mixed race	403 (3.9)	1740 (6.4)	1503 (6.2)	321 (3.8)
Black	705 (6.8)	1638 (6.0)	5423 (22.4)	310 (3.7)
Other	971 (9.4)	938 (3.5)	621 (2.6)	383 (4.6)
Missing	115 (1.1)	222 (0.8)	156 (0.6)	68 (0.8)
Gender				
Female	7023 (67.9)	17 446 (64.3)	17 697 (73.1)	5631 (67.0)
Male	3318 (32.1)	9645 (35.6)	6505 (26.9)	2762 (32.9)
Missing	9 (0.1)	35 (0.1)	15 (0.1)	12 (0.1)
IMD decile				
1 (Most deprived)	1305 (12.6)	2068 (7.6)	2256 (9.3)	399 (4.7)
2	2590 (25.0)	6371 (23.5)	6037 (24.9)	1006 (12.0)
3	2095 (20.2)	4696 (17.3)	4438 (18.3)	1136 (13.5)
4	1474 (14.2)	3274 (12.1)	2929 (12.1)	1198 (14.3)
5	1052 (10.2)	2466 (9.1)	2234 (9.2)	1102 (13.1)
6	817 (7.9)	2805 (10.3)	1975 (8.2)	1203 (14.3)
7	421 (4.1)	2126 (7.8)	1530 (6.3)	892 (10.6)
8	311 (3.0)	2044 (7.5)	1573 (6.5)	747 (8.9)
9	135 (1.3)	690 (2.5)	621 (2.6)	389 (4.6)
10 (Least deprived)	61 (0.6)	259 (1.0)	303 (1.3)	179 (2.1)
Missing	89 (0.9)	327 (1.2)	321 (1.3)	154 (1.8)
Employment status				
Employed	3648 (35.2)	16 333 (60.2)	13 376 (55.2)	4668 (55.5)
Unemployed	522 (5.0)	1541 (5.7)	1169 (4.8)	458 (5.4)
Student	873 (8.4)	1861 (6.9)	1173 (4.8)	502 (6.0)
Long-term ill	1916 (18.5)	2637 (9.7)	2600 (10.7)	625 (7.4)
Homemaker	1218 (11.8)	1129 (4.2)	1004 (4.1)	392 (4.7)
Not seeking work	1823 (17.6)	2495 (9.2)	2460 (10.2)	818 (9.7)
Volunteer	53 (0.5)	126 (0.5)	165 (0.7)	67 (0.8)
Retired	216 (2.1)	831 (3.1)	2075 (8.6)	808 (9.6)
Missing	81 (0.8)	173 (0.6)	195 (0.8)	67 (0.8)
Sexual orientation				
Heterosexual	9816 (94.8)	24 074 (88.8)	22 892 (94.5)	7799 (92.8)
Gay/lesbian	90 (0.9)	1009 (3.7)	364 (1.5)	135 (1.6)
Bisexual	35 (0.3)	735 (2.7)	195 (0.8)	125 (1.5)
Missing	409 (4.0)	1308 (4.8)	766 (3.22)	346 (4.1)
Clinical measures pretreatment, preexisting conditions and medication				
Depression symptoms pretreatment (PHQ-9), mean (SD)	17.0 (5.9)	14.8 (6.0)	15.0 (6.2)	14.5 (6.3)
Anxiety symptoms pretreatment (GAD-7), mean (SD)	15.0 (4.8)	13.4 (5.0)	13.6 (5.0)	13.3 (5.2)
WSAS–home, mean (SD)	4.3 (2.6)	3.6 (2.5)	3.6 (2.5)	3.6 (2.5)
WSAS–social, mean (SD)	4.9 (2.6)	4.5 (2.5)	4.5 (2.6)	4.2 (2.6)
WSAS–relationships, mean (SD)	4.5 (2.7)	3.7 (2.6)	3.7 (2.7)	3.6 (2.7)
WSAS–leisure, mean (SD)	4.4 (2.6)	4.2 (2.5)	4.0 (2.6)	4.0 (2.6)
Psychotropic medication				
Not prescribed	4726 (45.7)	14 466 (53.3)	12 388 (51.2)	4708 (56.0)
Prescribed and taking	4361 (42.1)	9522 (35.1)	9008 (37.2)	2739 (32.6)
Prescribed not taking	477 (4.6)	1294 (4.8)	1192 (4.9)	379 (4.5)
Missing	786 (7.6)	1844 (6.8)	1629 (6.7)	579 (6.9)
Self-reported long-term condition				
No	5809 (56.1)	17 601 (64.9)	13 966 (57.7)	4677 (55.6)
Yes	3070 (29.7)	6679 (24.6)	7551 (31.2)	2722 (32.4)
Missing	1471 (14.2)	2846 (10.5)	2700 (11.1)	1006 (12.0)
Treatment factors				
Diagnosis category				
Depression	5421 (52.4)	12 930 (47.7)	11 824 (48.8)	4578 (54.5)
GAD	990 (9.6)	4076 (15.0)	3192 (13.2)	1157 (13.8)
Mixed anxiety and depression	827 (8.0)	2100 (7.7)	1872 (7.7)	520 (6.2)
OCD	179 (1.7)	555 (2.0)	423 (1.7)	144 (1.7)
PTSD	695 (6.7)	809 (3.0)	898 (3.7)	225 (2.7)
Phobia and panic	788 (7.6)	2734 (10.1)	2096 (8.7)	501 (6.0)
Anxiety disorder not otherwise specified	371 (3.6)	682 (2.5)	724 (3.0)	250 (3.0)
Other	126 (1.2)	390 (1.4)	496 (2.0)	100 (1.2)
Missing	953 (9.2)	2850 (10.5)	2692 (11.1)	930 (11.1)
No. of sessions, mean (SD)	7.2 (4.6)	7.7 (4.8)	7.7 (4.8)	7.7 (4.8)
No. of canceled sessions, mean (SD)	1.5 (1.7)	1.4 (1.6)	1.4 (1.6)	1.5 (1.7)
Time between referral and assessment, mean (SD), wk	4.6 (9.4)	3.4 (7.1)	3.8 (7.6)	4.0 (7.0)
Time between assessment and treatment, mean (SD), wk	10.9 (9.8)	9.0 (8.7)	9.1 (9.0)	10.7 (9.2)

Abbreviations: IMD, index of multiple deprivation; GAD, generalized anxiety disorder; OCD, obsessive compulsive disorder; PTSD, posttraumatic stress disorder; PHQ, Patient Health Questionnaire; WSAS, Work and Social Adjustment Scale.

^a^ One-way analysis of variance was used for continuous variables and χ^2^ tests were used for categorical variables to test for differences in patient characteristics across the religion categories; all *P* values were less than .001, so they are not presented in this table.

Logistic regression models were constructed for each outcome. Model 1 investigated the unadjusted association between self-identified religion and outcomes with Muslim as the reference category. Following this, to investigate whether associations were independent of potential confounders, variables were added to the model sequentially. Model 2 additionally adjusted for treatment factors (number of sessions attended, number of sessions canceled, weeks from referral to assessment, and weeks from assessment to first session). Model 3 additionally adjusted for clinical factors related to treatment in NHS TTad services (PHQ-9 score, GAD-7 score, phobic anxiety scale items, diagnosis, functional impairment [Work and Social Adjustment Scale (WSAS) items]). Model 4 additionally adjusted for sociodemographic factors (age, gender, ethnicity, deprivation, long-term health condition, sexual orientation, employment status, and medication status).

To examine any changes in outcomes across religions over time, outcomes were also reported separately by 3 categories of year of first treatment appointment (2011-2014, 2015-2017, and 2018-2020). To investigate any effect modification by ethnicity, ethnicity was added as an interaction term to the fully adjusted model (model 4). Finally, patients belonging to the other religion category were further subdivided to examine treatment outcomes in patients self-identifying as Buddhist, Hindu, Jewish, and Sikh compared with Muslim separately, although their interaction with ethnicity could not be investigated due to the small sample sizes and most individuals being from a single ethnic background in these more granular religion categories.

One-way analysis of variance was used for continuous variables and χ^2^ tests were used for categorical variables to test for differences in patient characteristics across the religion categories. All tests were 2-tailed and *P* < .05 was considered statistically significant. Data were analyzed from September 2023 to October 2024 using Stata version 18 (StataCorp).

## Results

### Participant Characteristics

Of the 75 058 individuals referred to the 5 NHS TTad services from 2011 to 2020 who completed a course of psychological treatment, a total of 70 098 patients with data on self-reported religion (93.4%) were included in the analyses (mean [SD] age at referral, 39.2 [14.1] years; 47 797 [68.2%] female) (eFigure in Supplement 1). Of these patients, 10 350 (14.8%) self-identified as Muslim, 27 126 (38.7%) as having no religion, 24 217 (34.6%) as Christian, and 8405 (12.0%) identified with other religions. Those identifying as Muslim were most likely to identify as ethnically Asian (4876 participants [47.1%]), while patients of all other religion groups were most likely to identify as White with no religion (21 337 [78.7%]), Christian (15 920 [65.7%]), or other religion (3909 [46.5%]) (Table 1). Those who reported belonging to other religions (which includes Buddhism, Hinduism, Judaism, Sikhism, and others) also had a relatively high proportion of individuals identifying as ethnically Asian (3414 participants [40.6%]).

Pretreatment, those identifying as Muslim were most likely to live in deprived neighborhoods and be unemployed; 3895 Muslim patients (37.6%) were living in the most deprived quintile of areas in England (vs 8349 people with no religion [31.1%] and 8293 [34.2%] Christian patients), and 4261 (41.1%) were unemployed, long-term ill, or not seeking work (vs 6673 people with no religion [24.6%] and 6229 [25.7%] Christian patients). Muslim patients also had the highest average depression (PHQ-9), anxiety (GAD-7), and impairments in social functioning (WSAS) scores before treatment. Approximately 4838 Muslim patients (46.7%) reported being prescribed psychotropic medication, compared with 10 816 people with no religion (39.9%) and 10 200 Christian patients (42.1%). On average, Muslim patients had the longest wait times for both assessment and first treatment sessions and attended fewer sessions than those in any of the other groups.

### Association Between Religion and Treatment Outcomes

#### Primary Outcome

Muslim patients had the lowest likelihood of reliable recovery regardless of adjustment for potential confounders, although adjustment reduced inequalities in outcomes (Table 2). In the unadjusted model, the odds of reliable recovery were 91% to 94% higher in those who reported no religion, other religion, or Christian compared with Muslim patients. After full adjustment, the odds of reliable recovery were still higher in patients who did not self report any religion (odds ratio [OR], 1.34; 95% CI, 1.26-1.42) or self-reported Christian (OR, 1.39; 95% CI, 1.31-1.48) and other religion (OR, 1.25; 95% CI, 1.17-1.34) compared with Muslim patients.

**Table 2.** Associations Between Self-Identified Religion and Psychological Treatment Outcomes

Outcome	No. (%) with outcome	OR (95% CI)	
		Unadjusted model (model 1)	Fully adjusted model (model 4)^a^
Primary			
Reliable recovery			
Muslim	3113 (31.68)	1 [Reference]	1 [Reference]
No religion	11 689 (46.91)	1.91 (1.81-2.00)	1.34 (1.26-1.42)
Christian	10 496 (47.32)	1.94 (1.84-2.04)	1.39 (1.31-1.48)
Other religion	3548 (46.92)	1.91 (1.79-2.03)	1.25 (1.17-1.34)
Secondary			
Recovery			
Muslim	3258 (33.15)	1 [Reference]	1 [Reference]
No religion	12 261 (49.21)	1.95 (1.86-2.05)	1.33 (1.25-1.42)
Christian	10 968 (49.45)	1.97 (1.88-2.07)	1.39 (1.30-1.48)
Other religion	3755 (49.66)	1.99 (1.87-2.12)	1.26 (1.18-1.35)
Reliable improvement			
Muslim	5942 (57.41)	1 [Reference]	1 [Reference]
No religion	17 876 (65.90)	1.43 (1.37-1.50)	1.30 (1.23-1.38)
Christian	16 082 (66.41)	1.47 (1.40-1.54)	1.34 (1.26-1.42)
Other religion	5510 (65.56)	1.41 (1.33-1.50)	1.24 (1.16-1.33)
Reliable deterioration			
Muslim	1196 (11.56)	1 [Reference]	1 [Reference]
No religion	2088 (7.70)	0.64 (0.59-0.69)	0.71 (0.65-0.78)
Christian	1974 (8.15)	0.68 (0.63-0.73)	0.73 (0.67-0.80)
Other religion	645 (7.67)	0.64 (0.58-0.70)	0.71 (0.64-0.79)
Dropout			
Muslim	3369 (37.03)	1 [Reference]	1 [Reference]
No religion	8121 (32.73)	0.83 (0.79-0.87)	1.11 (1.04-1.19)
Christian	6792 (30.84)	0.76 (0.72-0.80)	1.09 (1.02-1.17)
Other religion	2141 (28.36)	0.67 (0.63-0.72)	1.03 (0.95-1.12)

Abbreviation: OR, odds ratio.

^a^ Model 4: model 1 adjusted for treatment factors (number of sessions attended, number of sessions canceled, weeks from referral to assessment, and weeks from assessment to first session), clinical factors related to National Health Service Talking Therapies treatment (Patient Health Questionnaire-9 score, Generalized Anxiety Disorder-7 score, phobic scale items, diagnosis category, and personal functioning [Work and Social Adjustment Scale items]), and sociodemographic and other pretreatment factors (age, gender, ethnicity, deprivation, long-term health condition, sexual orientation, employment status, and medication status).

**Figure 1.**
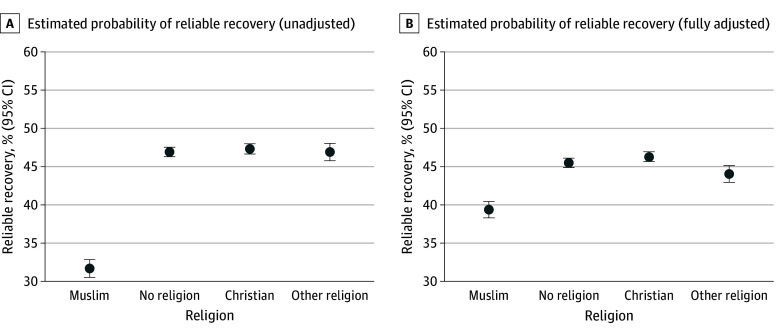
Estimated Probability of Reliable Recovery Across Religions in the Unadjusted and Fully Adjusted Models Predicted probabilities of patients achieving reliable recovery in each group were obtained using the relevant logistic regression models. The fully adjusted model (model 4) was adjusted for treatment factors (number of sessions attended, number of sessions canceled, weeks from referral to assessment, and weeks from assessment to first session), clinical factors related to treatment in National Health Service Talking Therapies for anxiety and depression services (Patient Health Questionnaire-9 score, Generalized Anxiety Disorder-7 score, phobic anxiety scale items, diagnosis, and functional impairment [Work and Social Adjustment Scale items]), and sociodemographic and other pretreatment factors (age, gender, ethnicity, deprivation, long-term health condition, sexual orientation, employment status, and medication status).

The estimated probability of reliable recovery among those identifying as Muslim was 39.4% (95% CI, 38.3-40.4) in the fully adjusted model (Figure 1). Christian patients had the highest estimated probability of reliable recovery among the groups (46.3%; 95% CI, 45.7-47.0), followed closely by those with no religion (45.5%; 95% CI, 44.9-46.1), and then by those who reported other religions (44.0%; 95% CI, 42.9-45.1). There were no significant differences in reliable recovery among patients who reported no religion, Christian, and other religion (eTable 4 in Supplement 1).

#### Secondary Outcomes

The patterns of association of religion with recovery and reliable improvement were similar to those for reliable recovery (Table 2). The likelihood of reliable deterioration was 27% to 29% lower in patients in the other 3 religion groups than in Muslim patients after full adjustment. The likelihood of dropout from treatment was also lower in the other 3 groups compared with Muslim patients in the unadjusted model, but this association was completely attenuated or reversed in the fully adjusted model.

### Changes in Associations Over Time

**Figure 2.**
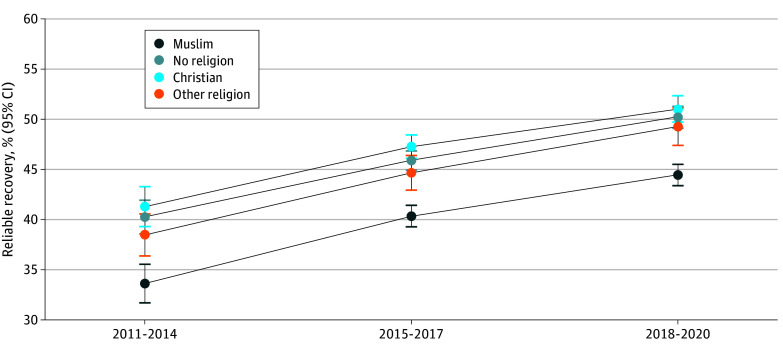
Estimated Probability of Reliable Recovery by Religion Over Time in the Fully Adjusted Model Predicted probabilities of patients achieving reliable recovery in each group were obtained using the relevant logistic regression models. Fully adjusted model (model 4): adjusted for treatment factors (number of sessions attended, number of sessions canceled, weeks from referral to assessment, and weeks from assessment to first session), clinical factors related to treatment in National Health Service Talking Therapies for anxiety and depression services (Patient Health Questionnaire-9 score, Generalized Anxiety Disorder-7 score, phobic anxiety scale items, diagnosis, and functional impairment [Work and Social Adjustment Scale items]), and sociodemographic and other pretreatment factors (age, gender, ethnicity, deprivation, long-term health condition, sexual orientation, employment status, and medication status).

Figure 2 shows the estimated probability of reliable recovery by religion across 3 categories of time, representing the years when the first treatment appointment occurred. The average probability of reliable recovery increased within all religion groups over time, and the disparity in reliable recovery across the groups has also narrowed with time. However, Muslim patients remained significantly less likely to reliably recover compared with patients in all other groups even in the most recent years (2018-2020). The findings were similar for the secondary outcomes (eTable 5 in Supplement 1), except for dropout, for which there were no significant differences across religion groups after full adjustment.

### Interaction Between Religion and Ethnicity

**Figure 3.**
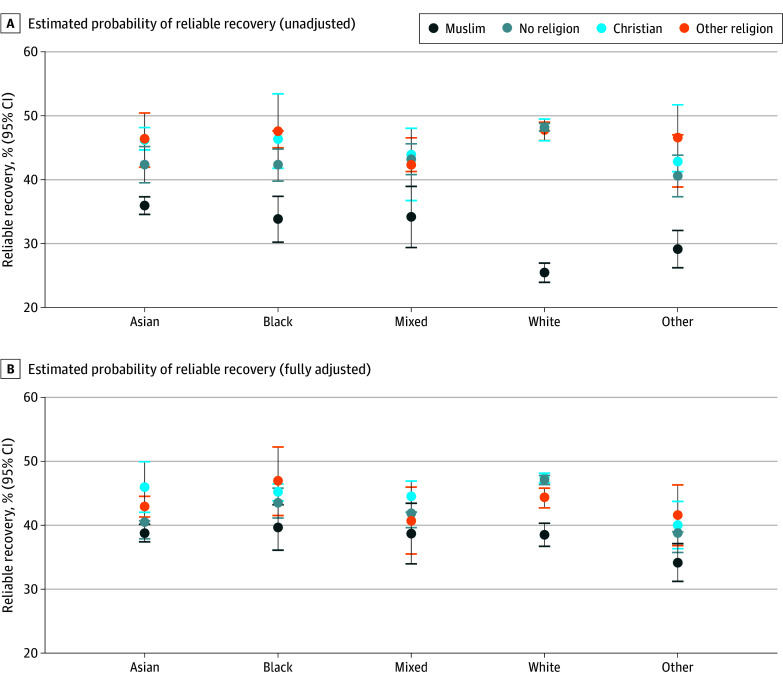
Estimated Probability of Reliable Recovery Across Religions by Intersections With Ethnicity in the Unadjusted and Fully Adjusted Models Predicted probabilities of patients achieving reliable recovery in each group were obtained using the relevant logistic regression models. Fully adjusted model (model 4) adjusted for treatment factors (number of sessions attended, number of sessions canceled, weeks from referral to assessment, and weeks from assessment to first session), clinical factors related to treatment in National Health Service Talking Therapies for anxiety and depression services (Patient Health Questionnaire-9 score, Generalized Anxiety Disorder-7 score, phobic anxiety scale items, diagnosis, functional impairment [Work and Social Adjustment Scale items]), and sociodemographic and other pretreatment factors (age, gender, ethnicity, deprivation, long-term health condition, sexual orientation, employment status, and medication status).

After full adjustment, there were interactions between religion and ethnicity for all outcomes (eTable 6 in Supplement 1). Muslim patients from White and other ethnic backgrounds were particularly less likely to reliably recover compared with any other combinations of religious-ethnic identity (Figure 3). Similar findings were observed for recovery and reliable improvement. Muslim patients from White ethnic backgrounds were also more likely to drop out compared with patients in any other group. Participant characteristics for Muslim patients of each ethnic group are reported in eTable 7 in Supplement 1.

### Sensitivity Analysis

There were no substantive differences in the findings using complete case analysis relative to the main analyses using multiply imputed data (eTable 8 in Supplement 1). All further subgroups within the other religion category (Jewish, Hindu, Sikh, Buddhist, and all other) had better therapy outcomes than Muslim patients in the unadjusted models (eTable 9 in Supplement 1). Many of the associations were attenuated after adjusting for clinical factors related to treatment in NHS TTad services (including baseline symptom severity) and sociodemographic and other pretreatment factors, but Muslim patients were still less likely to experience reliable recovery compared with all other subgroups except for Jewish patients, and were less likely to experience reliable improvement compared with all other subgroups including Jewish patients.

## Discussion

Despite substantial improvements in average treatment outcomes in NHS TTad services over time,^18,19,24^ Muslim patients still experienced significantly worse outcomes than patients in any other religion groups, including those with no religion. Previous studies have reported that Muslim patients are underrepresented in mental health services and have poor psychological treatment outcomes in countries where Muslim patients are minoritized, such as the UK and US.^14,15,25,26,27,28,29^ Although these studies rarely adjusted for important confounders, our study was able to demonstrate that inequalities in treatment outcomes by religion were somewhat attenuated but persist after adjustment for sociodemographic, treatment-related, and clinical characteristics. Moreover, although some studies suggest that Muslim patients are more likely to disengage with treatment, we found that adjusting for available confounders completely attenuated this association.

Studies have also found that Asian and minority ethnic patients experience poorer psychological treatment outcomes than the majority ethnic group.^25,26,30^ However, we found that Muslim patients of Asian, Black, and mixed race ethnicities had better outcomes compared with White Muslim patients, and that people of White and other ethnicities that did not identify as Muslim had better outcomes than people of these ethnicities that did. In an audit using locally collected data on nationality in 2 services (note nationality is not routinely recorded in NHS TTad services) we found that those who reported Turkish, Kurdish, Syrian, Iraqi, Albanian, or Kosovan nationality commonly identified as either ethnically White or using the other ethnicity category, and as Muslim. In the UK 2021 Census, proportions of individuals of these nationalities who live in the areas served by participating NHS TTad services approximately doubled compared with Census 2011.^31^ Therefore, data linkage with other health or statutory service datasets or routinely collecting data on nationality, asylum-seeker, and refugee status may help better understand the nature of the observed interaction effects found here.

Muslim patients had higher levels of symptom severity before treatment, which might indicate delays seeking therapy. As treatment outcomes are generally poorer for those with higher pretreatment severity and for those with longer episode durations,^32^ improving relationships with local referrers and to outreach in community and faith-based organizations is recommended. This can help facilitate referrals earlier in the course of the mental disorder and thus reduce the inequality in treatment outcomes. Currently, levels of engagement with faith-based community organizations vary across services, and further work is needed to investigate the impact of outreach activities on outcomes for Muslim patients.

Muslim patients, particularly those identifying as either ethnically White or of other ethnicities, were the most likely to be unemployed of all patient subgroups. Unemployment is strongly associated with poorer treatment outcomes^33,34,35^ and NHS TTad services have recently invested in employment advisory support to be offered to some patients alongside their psychological therapies. Offering such support routinely to those in particular subgroups has been found to double the rate of reliable recovery.^36^ There may be value in routinely offering employment support to Muslim patients that identify as ethnically White or of other ethnicities and who are either unemployed or report their employment to be at risk.

Data completion of self-identified religion, which is not a mandatory data item in NHS TTad, is key to monitoring and addressing inequalities. Further information, such as how much faith affects patients’ understanding of mental health problems, as well as data on refugee or asylum-seeker status, may further elucidate inequalities and how to address them.

### Limitations

This study had limitations. The findings of this study may not be generalizable to other clinical settings or different geographical areas, as the data were only collected from services within London. Variations in population demographics, staff diversity, and organizational features in other services may lead to different effects. Nevertheless, since there is a legal requirement of health care services in the UK to record information on religion for all patients who are willing to give it, inequalities identified by self-reported religion could be a measure with potential generalizability at the national level.

Our study was restricted to information routinely collected by NHS TTad services, excluding potentially important confounders such as religiosity, prior or concurrent support from a faith leader or healer, the religious identity of the therapists, and any adaptations made to therapy to account for the patient’s religious identity. There may also be residual confounding due to imperfectly measured covariates; for instance, the presence of any long-term health conditions may not be able to fully account for the burden of comorbidities. In addition, although most symptom measures are validated in a variety of languages, not all measures were validated in all languages, which may have led to some measurement error.

## Conclusion

In this retrospective cohort study, Muslim patients were less likely than patients of other religions or with no religion to achieve reliable recovery after psychological therapy; results varied by patient ethnicity. Clinical guidance has promoted the use of culturally adapted approaches for people who belong to minoritized ethnic groups.^13^ This includes culturally adapted therapy, staff training, and community outreach. Although this may inform some of the changes needed to reduce inequalities in treatment outcomes by religion, our findings highlight a need for further development to fully encompass religious identity and the intersectionality with ethnicity.

## Notes

Supplement 1. **eTable 1.** 2021 Census data on religious and ethnic diversity in the regions in which the participating NHS Talking Therapies services were located **eFigure. **Study participant flow diagram **eTable 2.** Available data and measures **eTable 3. **Clinical thresholds for Anxiety Disorder Specific Measures (ADSMs) **eTable 4.** Associations between religion and treatment outcomes **eTable 5.** Associations between religion and treatment outcomes by year of first treatment **eTable 6.** Interaction between religion and ethnicity: associations stratified by ethnicity **eTable 7.** Participant characteristics of Muslim Patients of each ethnic group **eTable 8.** Complete case analysis results **eTable 9.** Sensitivity analyses with more granular other religion categories
eReferences.

Supplement 2.
Data Sharing Statement
