# Supplementary material for: Psychological Therapy Outcomes and Engagement in People of Different Religions
Source: JAMA Netw Open. 2025 Apr 8;8(4):e254026. doi: 10.1001/jamanetworkopen.2025.4026 (PMC11979733; doi:10.1001/jamanetworkopen.2025.4026)
Supplement: Supplement 1. — eTable 1. 2021 Census data on religious and ethnic diversity in the regions in which the participating NHS Talking Therapies services were located eFigure. Study participant flow diagram eTable 2. Available data and measures eTable 3. Clinical thresholds for Anxiety Disorder Specific Measures (ADSMs) eTable 4. Associations between religion and treatment outcomes eTable 5. Associations between religion and treatment outcomes by year of first treatment eTable 6. Interaction between religion and ethnicity: associations stratified by ethnicity eTable 7. Participant characteristics of Muslim Patients of each ethnic group eTable 8. Complete case analysis results eTable 9. Sensitivity analyses with more granular other religion categories eReferences. [file jamanetwopen-e254026-s001.pdf]

Supplemental Online Content

Shafan-Azhar Z, Suh JW, Delamain H, et al. Psychological therapy outcomes and engagement in people of different religions in England. *JAMA Netw Open*. 2025;8(4):e254026. doi:10.1001/jamanetworkopen.2025.4026

eTable 1. 2021 Census data on religious and ethnic diversity in the regions in which the participating NHS Talking Therapies services were located

eFigure. Study participant flow diagram

eTable 2. Available data and measures

eTable 3. Clinical thresholds for Anxiety Disorder Specific Measures (ADSMs)

eTable 4. Associations between religion and treatment outcomes

eTable 5. Associations between religion and treatment outcomes by year of first treatment

eTable 6. Interaction between religion and ethnicity: associations stratified by ethnicity

eTable 7. Participant characteristics of Muslim Patients of each ethnic group

eTable 8. Complete case analysis results

eTable 9. Sensitivity analyses with more granular other religion categories

eReferences

© 2025 Shafan-Azhar Z et al. *JAMA Network Open*.

This supplemental material has been provided by the authors to give readers additional information about their work.

© 2025 Shafan-Azhar Z et al. *JAMA Network Open*.

A) Further information on data source and participants

eTable 1. 2021 Census data on religious and ethnic diversity in the regions in which the participating NHS Talking Therapies services were located.

Religion

**geography**	Barnet		Enfield		Haringey		Barking and Dagenham		Redbridge		London		England	
**measures**	N	%	N	%	N	%	N	%	N	%	N	%	N	%
**Religion**														
Total: All usual residents	389,344	100	329,984	100	264,238	100	218,867	100	310,260	100	8,799,728	100	56,490,048	100
No religion	78,684	20	65,241	20	83,535	32	41,191	19	38,999	13	2,380,404	27	20,715,664	37
Christian	142,321	37	153,015	46	103,944	39	99,342	45	94,473	30	3,577,681	41	26,167,899	46
Buddhist	4,158	1	1,716	1	2,455	1	821	0	1,611	1	77,425	1	262,433	0
Hindu	22,105	6	10,231	3	3,529	1	6,596	3	34,372	11	453,034	5	1,020,533	2
Jewish	56,616	15	3,713	1	9,397	4	272	0	6,412	2	145,466	2	269,283	0
Muslim	47,688	12	61,477	19	33,295	13	53,389	24	97,068	31	1,318,754	15	3,801,186	7
Sikh	1,524	0	1,199	0	892	0	4,284	2	17,622	6	144,543	2	520,092	1
Other religion	5,192	1	10,351	3	6,164	2	981	0	2,028	1	86,759	1	332,410	1
Not answered	31,056	8	23,041	7	21,027	8	11,991	5	17,675	6	615,662	7	3,400,548	6

In order to protect against disclosure of personal information, records have been swapped between different geographic areas and counts perturbed by small amounts. Small counts at the lowest geographies will be most affected.

Definition: The religion people connect or identify with (their religious affiliation), whether or not they practise or have belief in it. This question was voluntary and includes people who identified with one of eight tick-box response options, including "No religion", alongside those who chose not to answer this question.

Comparability with 2011: Broadly comparable. This derived variable can be generally compared with the same variable used in the 2011 Census, but there are some quality issues in the data.

© 2025 Shafan-Azhar Z et al. *JAMA Network Open*.

Ethnicity

**geography**	Barnet		Enfield		Haringey		Barking and Dagenham		Redbridge		London		England	
**measures**	N	%	N	%	N	%	N	%	N	%	N	%	N	%
**Ethnic group**														
Total: All usual residents	389,344	100	329,985	100	264,238	100	218,871	100	310,261	100	8,799,725	100	56,490,048	100
Asian, Asian British or Asian Welsh	74,972	19	37,973	12	23,080	9	56,583	26	146,833	47	1,817,640	21	5,426,392	10
Black, Black British, Black Welsh, Caribbean or African	30,651	8	60,512	18	46,466	18	46,807	21	26,096	8	1,188,370	14	2,381,724	4
Mixed or Multiple ethnic groups	20,889	5	19,558	6	18,556	7	9,320	4	12,736	4	505,775	6	1,669,378	3
White	224,762	58	171,884	52	150,581	57	98,275	45	107,974	35	4,731,172	54	45,783,401	81
Other ethnic group	38,070	10	40,058	12	25,555	10	7,886	4	16,622	5	556,768	6	1,229,153	2

In order to protect against disclosure of personal information, records have been swapped between different geographic areas and counts perturbed by small amounts. Small counts at the lowest geographies will be most affected.

Description: The ethnic group that the person completing the census feels they belong to. This could be based on their culture, family background, identity or physical appearance. Respondents could choose one out of 19 tick-box response categories, including write-in response options.

Comparability with 2011: Broadly comparable. The question about the ethnic group people feel they belong to is self-identified and is subjectively meaningful to the person answering the question. This means that how a person chooses to identify can change over time.
We have included a new Roma category next to the Gypsy or Irish Traveller tick-box within the White category. We have also added a write-in option for those selecting African within the Black, Caribbean or Black British category. This means that a more specific ethnic background could be recorded.

Source: ONS - 2021 Census

© 2025 Shafan-Azhar Z et al. *JAMA Network Open*.

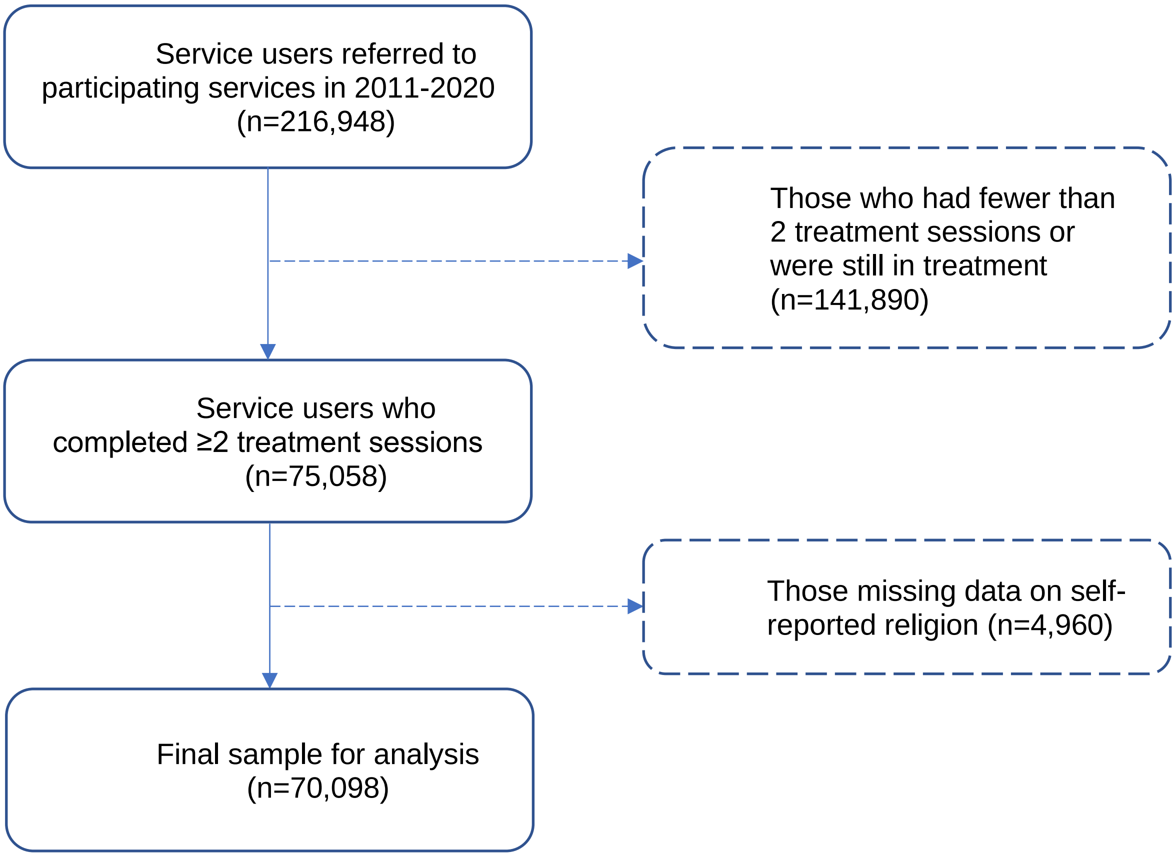

eFigure 1. Study participant flow diagram

© 2025 Shafan-Azhar Z et al. *JAMA Network Open*.

eTable 2. Available data and measures

Data Item	Questionnaire	Information on measurement
Exposure & Effect modifier		
Religion	N/A	Religion was self-reported, respondents have an option of 158 different faiths and denominations which include broad categories e.g. (Christian or Muslim) and specific denominations (including 80 options for Christians e.g. 'Church of England' or 'Roman Catholic', and eight for Muslims e.g. 'Shi'ite', or 'Sunni'). These were categorized into the most prevalent higher-order categories in the services from which data were collected: 1) No religion, 2) Christian, 3) Muslim, and 4) Other (Buddhist, Hindu, Jewish, Sikh, Other).
Ethnicity	N/A	Ethnicity was self-reported at the point of referral using two linked lists of options. The first includes higher-order categories (e.g. Asian or Asian British, Black or Black British, Mixed ethnicity, White, Other ethnic groups) and a second list of sub-categories (e.g. Asian – Indian, Asian – Pakistani, Asian – Bangladeshi, Any other Asian background). In our study, ethnicity was categorized into 'White', 'Mixed', 'Asian', 'Black', and 'Other' in accordance with the Office of National Statistics (ONS)'s higher-order categorization list used in UK Census.<br><br>The term Asian was used to connote patients that identified as Asian or Asian British in the higher-order category list and then as either Indian, Pakistani, Bangladeshi, or 'Any other Asian background' in the sub-category list. Note Chinese is not included on the sub-category list in the Asian higher-order category, but instead in the Other higher-order category. The term Black was used to connote those identifying as Black or Black British in the higher-order category and then Caribbean, African or 'Any other Black background' in the sub-category list. Mixed ethnicity included those identifying as Mixed in the higher-order category list and then Mixed White and Asian, Mixed White and Black Caribbean, Mixed White and Black African, or 'Any other mixed ethnicity background'. White included those identifying as White in the higher-order list then as any of British, Irish, or 'Any other White background'. Other ethnicities included those identifying as Other in the higher-order category and then as either Chinese or of Any other ethnic group in the sub-category.
Clinical characteristics		
Anxiety	The Generalized Anxiety Disorder Scale 7-item	For general anxiety disorder, scoring above 8 is used to indicate caseness, and reductions of

© 2025 Shafan-Azhar Z et al. *JAMA Network Open*.

	version (GAD-7) (Spitzer et al., 2006)	scores by 4 or higher indicate reliable improvement. For alternative anxiety disorders, ‘anxiety disorder-specific measures’ are used. These are measures specifically related to the type of anxiety disorder. Improvement is represented by their Anxiety-Disorder Specific Measure (ADSM) for specific anxiety disorders improving according to the error of measurement (Saunders et al., 2021).
Depression	Patient Health Questionnaire 9-item (PHQ-9) (Kroenke et al., 2001)	Scores of 10 and above are suggestive of clinical caseness for depression and reductions of 6 or more indicate reliable improvement.
Diagnosis category	N/A	Diagnosis category (based on ICD-10 codes) relates to the agreed focus of psychological treatment, and is recorded by the treating clinician after discussion with the patient. NHS Talking Therapies services offer treatment in accordance with clinical guidelines for particular diagnoses issued by the National Institute for Health and Care Excellence. This means that the recorded diagnosis informs clinical decision making on which evidence-based treatment to offer to the patient. Diagnosis categories included depression (depressive episode, recurrent depression), mixed anxiety & depression, GAD, obsessive-compulsive disorder, post-traumatic stress disorder, phobic anxiety and panic, unspecified anxiety disorder, and other diagnoses (alcohol related mental or behavioral disorder, bereavement, bipolar affective disorder, eating disorder, hypochondriacal disorder, somatoform disorder, etc.).
Phobic Anxiety	The IAPT Phobia Scales (National IAPT Programme Team, 2011)	Three questions are included, and each relate to a different type of phobic anxiety. These are agoraphobia, social phobia, and specific phobia.
Personal Functioning	The Work and Social Adjustment Scale (WSAS) (Mundt et al., 2002)	This scale measures the personal functioning of individuals in a range of domains. These include work, home, social, relationships, and leisure. Each domain score ranges from 0-8. As the work variable can often be recorded as ‘not applicable’ for those not in work, this variable was excluded from the analysis.
Medication	N/A	These are recorded as: ‘prescribed and taking’, ‘prescribed and not taking’ and ‘not prescribed’. This is recorded at each point of clinical contact.

Socio-demographics

Demographics	N/A	Included in the dataset were (at the point of referral): age, self-reported gender (female, male), sexual orientation (heterosexual, gay/lesbian, bisexual), and index of multiple deprivation (IMD) ranks and deciles. Index of multiple deprivations includes ranks that rank the level of deprivation of certain geographical areas, and these are split into

© 2025 Shafan-Azhar Z et al. *JAMA Network Open*.

		deciles. For example, the most deprived 10% of areas is the first decile (1).
Employment Status	N/A	Patients select their employment status from the following: ‘Employed’, ‘Unemployed’, ‘Student’, ‘Long-term sick’, ‘Homemaker’, ‘Not seeking work’, ‘Volunteer’, ‘Retired.’
Long-term health conditions	N/A	Patients are asked whether they have a long-term physical health condition (yes, no).

Treatment and Service factors

Treatment factors	N/A	This included:<br>The total number of sessions attended.<br>The total number of sessions cancelled.<br>The number of weeks passed between referral and assessment, and between assessment and first treatment sessions.

© 2025 Shafan-Azhar Z et al. *JAMA Network Open*.

eTable 3. Clinical thresholds for Anxiety Disorder Specific Measures (ADSMs)

Anxiety disorder	Recommended ADSM	Threshold for caseness	Threshold for reliable improvement
Agoraphobia	Mobility Inventory [1]	2.3	0.73
Health anxiety	Health Anxiety Inventory [2]	18	4
Obsessive compulsive disorder (OCD)	Obsessive Compulsive Inventory [3]	40	32
Panic disorder	Panic Disorder Severity Scale [4]	8	5
Post-traumatic stress disorder (PTSD)	Impact of Events Scale (IES-R) [5][until October 2020]	33	9
	PTSD Checklist for DSM-5 (PCL-5) [6]	32	10
Social anxiety disorder	Social Phobia Inventory [7]	19	10

© 2025 Shafan-Azhar Z et al. *JAMA Network Open*.

eTable 4. Associations between religion and treatment outcomes

Outcomes	Number of events in the final sample for analysis (N=70,098)
**PRIMARY OUTCOME**	
**Reliable recovery**	
Muslim	3,113
No religion	11,689
Christian	10,496
Other religion	3,548
**SECONDARY OUTCOMES**	
**Recovery**	
Muslim	3,258
No religion	12,261
Christian	10,968
Other religion	3,755
**Reliable improvement**	
Muslim	5,942
No religion	17,876
Christian	16,082
Other religion	5,510
**Reliable deterioration**	
Muslim	1,196
No religion	2,088
Christian	1,974
Other religion	645
**Dropout**	
Muslim	3,369
No religion	8,121
Christian	6,792
Other religion	2,141

© 2025 Shafan-Azhar Z et al. *JAMA Network Open*.

Muslim as the reference group	Model 1	Model 2	Model 3	Model 4
	Unadjusted	Adjusted for treatment factors	Model 2 additionally adjusted for clinical factors	Model 3 additionally adjusted for socio-demographic factors
	OR (95% CI)	OR (95% CI)	OR (95% CI)	OR (95% CI)
**PRIMARY OUTCOME**				
**Reliable recovery**				
Muslim	Referent	Referent	Referent	Referent
No religion	1.91 (1.81; 2.00)	1.81 (1.72; 1.91)	1.45 (1.37; 1.53)	1.34 (1.26; 1.42)
Christian	1.94 (1.84; 2.04)	1.84 (1.75; 1.93)	1.54 (1.46; 1.63)	1.39 (1.31; 1.48)
Other religion	1.91 (1.79; 2.03)	1.84 (1.73; 1.96)	1.45 (1.36; 1.55)	1.25 (1.17; 1.34)
**SECONDARY OUTCOMES**				
**Recovery**				
Muslim	Referent	Referent	Referent	Referent
No religion	1.95 (1.86; 2.05)	1.86 (1.77; 1.96)	1.43 (1.36; 1.51)	1.33 (1.25; 1.42)
Christian	1.97 (1.88; 2.07)	1.87 (1.78; 1.97)	1.53 (1.45; 1.62)	1.39 (1.30; 1.48)
Other religion	1.99 (1.87; 2.12)	1.93 (1.81; 2.05)	1.47 (1.37; 1.57)	1.26 (1.18; 1.35)
**Reliable improvement**				
Muslim	Referent	Referent	Referent	Referent
No religion	1.43 (1.37; 1.50)	1.36 (1.30; 1.43)	1.43 (1.36; 1.50)	1.30 (1.23; 1.38)
Christian	1.47 (1.40; 1.54)	1.40 (1.34; 1.47)	1.48 (1.41; 1.56)	1.34 (1.26; 1.42)
Other religion	1.41 (1.33; 1.50)	1.35 (1.27; 1.43)	1.42 (1.33; 1.51)	1.24 (1.16; 1.33)
**Reliable deterioration**				
Muslim	Referent	Referent	Referent	Referent
No religion	0.64 (0.59; 0.69)	0.67 (0.62; 0.72)	0.61 (0.56; 0.66)	0.71 (0.65; 0.78)
Christian	0.68 (0.63; 0.73)	0.71 (0.66; 0.77)	0.64 (0.59; 0.70)	0.73 (0.67; 0.80)
Other religion	0.64 (0.58; 0.70)	0.66 (0.60; 0.73)	0.59 (0.53; 0.65)	0.71 (0.64; 0.79)
**Dropout**				
Muslim	Referent	Referent	Referent	Referent
No religion	0.83 (0.79; 0.87)	0.89 (0.84; 0.94)	1.05 (0.99; 1.12)	1.11 (1.04; 1.19)
Christian	0.76 (0.72; 0.80)	0.81 (0.76; 0.86)	0.94 (0.88; 1.00)	1.09 (1.02; 1.17)
Other religion	0.67 (0.63; 0.72)	0.70 (0.65; 0.76)	0.86 (0.79; 0.93)	1.03 (0.95; 1.12)

Model 2: Model 1 additionally adjusted for treatment factors (number of sessions attended, number of sessions cancelled, weeks from referral to assessment, weeks from assessment to first session). Model 3: Model 2 additionally adjusted for clinical factors (PHQ-9 score, GAD-7 score, phobic scale items, diagnosis category, personal functioning (WSAS items)). Model 4: Model 3 additionally adjusted for socio-demographic factors (age, gender, ethnicity, deprivation, long-term health condition, sexual orientation, employment status, medication status). OR=Odds Ratio, CI=Confidence Interval

© 2025 Shafan-Azhar Z et al. *JAMA Network Open*.

No religion as the reference group	Model 1	Model 2	Model 3	Model 4
	Unadjusted	Adjusted for treatment factors	Model 2 additionally adjusted for clinical factors	Model 3 additionally adjusted for socio-demographic factors
	OR (95% CI)	OR (95% CI)	OR (95% CI)	OR (95% CI)
**PRIMARY OUTCOME**				
**Reliable recovery**				
No religion	Referent	Referent	Referent	Referent
Muslim	0.52 (0.50; 0.55)	0.55 (0.52; 0.58)	0.69 (0.65; 0.73)	0.75 (0.70; 0.79)
Christian	1.02 (0.98; 1.05)	1.01 (0.98; 1.05)	1.07 (1.03; 1.11)	1.04 (1.00; 1.08)
Other	1.00 (0.95; 1.05)	1.02 (0.96; 1.07)	1.00 (0.95; 1.06)	0.93 (0.88; 0.99)
**SECONDARY OUTCOMES**				
**Recovery**				
No religion	Referent	Referent	Referent	Referent
Muslim	0.51 (0.49; 0.54)	0.54 (0.51; 0.57)	0.70 (0.66; 0.74)	0.75 (0.70; 0.80)
Christian	1.01 (0.97; 1.05)	1.01 (0.97; 1.04)	1.07 (1.03; 1.11)	1.04 (1.00; 1.08)
Other	1.02 (0.97; 1.07)	1.04 (0.98; 1.09)	1.02 (0.97; 1.08)	0.95 (0.89; 1.01)
**Reliable improvement**				
No religion	Referent	Referent	Referent	Referent
Muslim	0.70 (0.67; 0.73)	0.73 (0.70; 0.77)	0.70 (0.66; 0.74)	0.77 (0.72; 0.81)
Christian	1.02 (0.99; 1.06)	1.03 (0.99; 1.07)	1.04 (1.00; 1.08)	1.02 (0.98; 1.07)
Other	0.98 (0.94; 1.04)	0.99 (0.93; 1.04)	0.99 (0.94; 1.05)	0.95 (0.90; 1.01)
**Reliable deterioration**				
No religion	Referent	Referent	Referent	Referent
Muslim	1.57 (1.45; 1.69)	1.49 (1.39; 1.61)	1.65 (1.52; 1.79)	1.41 (1.29; 1.54)
Christian	1.06 (1.00; 1.13)	1.06 (1.00; 1.13)	1.06 (0.99; 1.13)	1.03 (0.96; 1.11)
Other	1.00 (0.91; 1.09)	0.99 (0.90; 1.08)	0.97 (0.88; 1.07)	1.00 (0.90; 1.11)
**Dropout**				
No religion	Referent	Referent	Referent	Referent
Muslim	1.21 (1.15; 1.27)	1.13 (1.06; 1.20)	0.95 (0.90; 1.01)	0.90 (0.84; 0.97)
Christian	0.92 (0.88; 0.95)	0.91 (0.87; 0.95)	0.90 (0.85; 0.94)	0.98 (0.94; 1.03)
Other	0.81 (0.77; 0.86)	0.79 (0.74; 0.85)	0.82 (0.77; 0.88)	0.93 (0.86; 1.00)

Model 2: Model 1 additionally adjusted for treatment factors (number of sessions attended, number of sessions cancelled, weeks from referral to assessment, weeks from assessment to first session). Model 3: Model 2 additionally adjusted for clinical factors (PHQ-9 score, GAD-7 score, phobic scale items, diagnosis category, personal functioning (WSAS items)). Model 4: Model 3 additionally adjusted for socio-demographic factors (age, gender, ethnicity, deprivation, long-term health condition, sexual orientation, employment status, medication status). OR=Odds Ratio, CI=Confidence Interval

© 2025 Shafan-Azhar Z et al. *JAMA Network Open*.

Christian as the reference group	Model 1	Model 2	Model 3	Model 4
	Unadjusted	Adjusted for treatment factors	Model 2 additionally adjusted for clinical factors	Model 3 additionally adjusted for socio-demographic factors
	OR (95% CI)	OR (95% CI)	OR (95% CI)	OR (95% CI)
**PRIMARY OUTCOME**				
**Reliable recovery**				
Christian	Referent	Referent	Referent	Referent
No religion	0.98 (0.95; 1.02)	0.99 (0.95; 1.02)	0.94 (0.90; 0.97)	0.96 (0.92; 1.00)
Muslim	0.52 (0.49; 0.54)	0.54 (0.52; 0.57)	0.65 (0.61; 0.68)	0.72 (0.68; 0.77)
Other	0.98 (0.93; 1.04)	1.00 (0.95; 1.06)	0.94 (0.89; 0.99)	0.90 (0.84; 0.95)
**SECONDARY OUTCOMES**				
**Recovery**				
Christian	Referent	Referent	Referent	Referent
No religion	0.99 (0.96; 1.03)	0.99 (0.96; 1.03)	0.93 (0.90; 0.97)	0.96 (0.92; 1.00)
Muslim	0.51 (0.48; 0.53)	0.53 (0.51; 0.56)	0.65 (0.62; 0.69)	0.72 (0.68; 0.77)
Other	1.01 (0.96; 1.06)	1.03 (0.98; 1.09)	0.96 (0.90; 1.01)	0.91 (0.86; 0.97)
**Reliable improvement**				
Christian	Referent	Referent	Referent	Referent
No religion	0.98 (0.94; 1.01)	0.97 (0.94; 1.01)	0.96 (0.93; 1.00)	0.98 (0.94; 1.02)
Muslim	0.68 (0.65; 0.71)	0.71 (0.68; 0.75)	0.67 (0.64; 0.71)	0.75 (0.71; 0.79)
Other	0.96 (0.91; 1.01)	0.96 (0.91; 1.01)	0.95 (0.90; 1.01)	0.93 (0.88; 0.99)
**Reliable deterioration**				
Christian	Referent	Referent	Referent	Referent
No religion	0.94 (0.88; 1.00)	0.94 (0.88; 1.00)	0.94 (0.88; 1.01)	0.97 (0.90; 1.04)
Muslim	1.47 (1.36; 1.59)	1.41 (1.30; 1.52)	1.56 (1.44; 1.68)	1.36 (1.24; 1.49)
Other	0.94 (0.85; 1.03)	0.93 (0.84; 1.02)	0.92 (0.83; 1.01)	0.97 (0.88; 1.07)
**Dropout**				
Christian	Referent	Referent	Referent	Referent
No religion	1.09 (1.05; 1.13)	1.10 (1.05; 1.15)	1.12 (1.07; 1.17)	1.02 (0.97; 1.07)
Muslim	1.32 (1.25; 1.39)	1.24 (1.16; 1.31)	1.07 (1.00; 1.13)	0.92 (0.85; 0.98)
Other	0.89 (0.84; 0.94)	0.87 (0.81; 0.93)	0.92 (0.86; 0.98)	0.95 (0.88; 1.02)

Model 2: Model 1 additionally adjusted for treatment factors (number of sessions attended, number of sessions cancelled, weeks from referral to assessment, weeks from assessment to first session). Model 3: Model 2 additionally adjusted for clinical factors (PHQ-9 score, GAD-7 score, phobic scale items, diagnosis category, personal functioning (WSAS items)). Model 4: Model 3 additionally adjusted for socio-demographic factors (age, gender, ethnicity, deprivation, long-term health condition, sexual orientation, employment status, medication status). OR=Odds Ratio, CI=Confidence Interval

© 2025 Shafan-Azhar Z et al. *JAMA Network Open*.

Other religion as the reference group	Model 1	Model 2	Model 3	Model 4
	Unadjusted	Adjusted for treatment factors	Model 2 additionally adjusted for clinical factors	Model 3 additionally adjusted for socio-demographic factors
	OR (95% CI)	OR (95% CI)	OR (95% CI)	OR (95% CI)
**PRIMARY OUTCOME**				
**Reliable recovery**				
Other	Referent	Referent	Referent	Referent
No religion	1.00 (0.95; 1.05)	0.98 (0.93; 1.04)	1.00 (0.95; 1.06)	1.07 (1.01; 1.14)
Christian	1.02 (0.96; 1.07)	1.00 (0.94; 1.05)	1.07 (1.01; 1.13)	1.11 (1.05; 1.18)
Muslim	0.52 (0.49; 0.56)	0.54 (0.51; 0.58)	0.69 (0.65; 0.74)	0.80 (0.75; 0.86)
**SECONDARY OUTCOMES**				
**Recovery**				
Other	Referent	Referent	Referent	Referent
No religion	0.98 (0.93; 1.03)	0.96 (0.92; 1.02)	0.98 (0.92; 1.03)	1.06 (0.99; 1.12)
Christian	0.99 (0.94; 1.04)	0.97 (0.92; 1.02)	1.05 (0.99; 1.11)	1.10 (1.03; 1.17)
Muslim	0.50 (0.47; 0.53)	0.52 (0.49; 0.55)	0.68 (0.64; 0.73)	0.79 (0.74; 0.85)
**Reliable improvement**				
Other	Referent	Referent	Referent	Referent
No religion	1.02 (0.96; 1.07)	1.01 (0.96; 1.07)	1.01 (0.96; 1.07)	1.05 (0.99; 1.11)
Christian	1.04 (0.99; 1.09)	1.04 (0.99; 1.10)	1.05 (0.99; 1.11)	1.07 (1.01; 1.14)
Muslim	0.71 (0.67; 0.75)	0.74 (0.70; 0.79)	0.71 (0.66; 0.75)	0.80 (0.75; 0.86)
**Reliable deterioration**				
Other	Referent	Referent	Referent	Referent
No religion	1.00 (0.92; 1.10)	1.02 (0.93; 1.11)	1.03 (0.94; 1.13)	1.00 (0.90; 1.11)
Christian	1.07 (0.97; 1.17)	1.08 (0.98; 1.18)	1.09 (0.99; 1.20)	1.03 (0.93; 1.14)
Muslim	1.57 (1.42; 1.74)	1.52 (1.37; 1.68)	1.70 (1.53; 1.88)	1.41 (1.26; 1.56)
**Dropout**				
Other	Referent	Referent	Referent	Referent
No religion	1.23 (1.16; 1.30)	1.26 (1.18; 1.35)	1.22 (1.14; 1.31)	1.07 (1.00; 1.16)
Christian	1.13 (1.06; 1.19)	1.15 (1.07; 1.23)	1.09 (1.02; 1.17)	1.06 (0.98; 1.14)
Muslim	1.49 (1.39; 1.59)	1.42 (1.32; 1.53)	1.16 (1.08; 1.26)	0.97 (0.89; 1.05)

Model 2: Model 1 additionally adjusted for treatment factors (number of sessions attended, number of sessions cancelled, weeks from referral to assessment, weeks from assessment to first session). Model 3: Model 2 additionally adjusted for clinical factors (PHQ-9 score, GAD-7 score, phobic scale items, diagnosis category, personal functioning (WSAS items)). Model 4: Model 3 additionally adjusted for socio-demographic factors (age, gender, ethnicity, deprivation, long-term health condition, sexual orientation, employment status, medication status). OR=Odds Ratio, CI=Confidence Interval

© 2025 Shafan-Azhar Z et al. *JAMA Network Open*.

eTable 5. Associations between religion and treatment outcomes by year of first treatment

First treatment in 2011-2014	Model 1	Model 2	Model 3	Model 4
	Unadjusted<br><br>OR (95% CI)	Adjusted for treatment factors<br><br>OR (95% CI)	Model 2 additionally adjusted for clinical factors<br><br>OR (95% CI)	Model 3 additionally adjusted for socio-demographic factors<br>OR (95% CI)
**PRIMARY OUTCOME**				
**Reliable recovery**				
Muslim	Referent	Referent	Referent	Referent
No religion	2.05 (1.86; 2.25)	1.95 (1.77; 2.15)	1.53 (1.38; 1.70)	1.40 (1.24; 1.57)
Christian	2.05 (1.86; 2.26)	1.93 (1.75; 2.13)	1.63 (1.47; 1.80)	1.47 (1.31; 1.65)
Other religion	2.07 (1.83; 2.34)	1.95 (1.72; 2.21)	1.47 (1.29; 1.68)	1.28 (1.11; 1.47)
**SECONDARY OUTCOMES**				
**Recovery**				
Muslim	Referent	Referent	Referent	Referent
No religion	2.08 (1.89; 2.29)	1.99 (1.81; 2.19)	1.53 (1.37; 1.69)	1.39 (1.24; 1.57)
Christian	2.07 (1.89; 2.28)	1.96 (1.78; 2.15)	1.62 (1.46; 1.79)	1.47 (1.30; 1.65)
Other religion	2.18 (1.93; 2.47)	2.06 (1.82; 2.34)	1.51 (1.32; 1.72)	1.31 (1.14; 1.51)
**Reliable improvement**				
Muslim	Referent	Referent	Referent	Referent
No religion	1.41 (1.30; 1.54)	1.35 (1.24; 1.47)	1.40 (1.28; 1.53)	1.24 (1.12; 1.37)
Christian	1.49 (1.37; 1.62)	1.41 (1.30; 1.54)	1.49 (1.36; 1.63)	1.31 (1.18; 1.44)
Other religion	1.39 (1.24; 1.56)	1.29 (1.15; 1.45)	1.39 (1.23; 1.57)	1.22 (1.08; 1.39)
**Reliable deterioration**				
Muslim	Referent	Referent	Referent	Referent
No religion	0.68 (0.60; 0.78)	0.71 (0.63; 0.81)	0.63 (0.55; 0.73)	0.76 (0.66; 0.89)
Christian	0.67 (0.59; 0.76)	0.70 (0.62; 0.80)	0.63 (0.55; 0.72)	0.72 (0.62; 0.84)
Other religion	0.66 (0.55; 0.79)	0.69 (0.58; 0.83)	0.59 (0.49; 0.71)	0.72 (0.59; 0.88)
**Dropout**				
Muslim	Referent	Referent	Referent	Referent
No religion	1.08 (0.98; 1.19)	1.08 (0.97; 1.21)	1.27 (1.14; 1.43)	1.32 (1.16; 1.50)
Christian	0.96 (0.87; 1.05)	0.96 (0.86; 1.07)	1.10 (0.99; 1.24)	1.29 (1.14; 1.46)
Other religion	0.70 (0.62; 0.80)	0.77 (0.66; 0.89)	0.96 (0.82; 1.12)	1.18 (1.01; 1.38)

Model 2: Model 1 additionally adjusted for treatment factors (number of sessions attended, number of sessions cancelled, weeks from referral to assessment, weeks from assessment to first session). Model 3: Model 2 additionally adjusted for clinical factors (PHQ-9 score, GAD-7 score, phobic scale items, diagnosis category, personal functioning (WSAS items)). Model 4: Model 3 additionally adjusted for socio-demographic factors (age, gender, ethnicity, deprivation, long-term health condition, sexual orientation, employment status, medication status). OR=Odds Ratio, CI=Confidence Interval

© 2025 Shafan-Azhar Z et al. *JAMA Network Open*.

First treatment in 2015-2017	Model 1	Model 2	Model 3	Model 4
	Unadjusted	Adjusted for treatment factors	Model 2 additionally adjusted for clinical factors	Model 3 additionally adjusted for socio-demographic factors
	OR (95% CI)	OR (95% CI)	OR (95% CI)	OR (95% CI)
**PRIMARY OUTCOME**				
**Reliable recovery**				
Muslim	Referent	Referent	Referent	Referent
No religion	1.95 (1.80; 2.10)	1.86 (1.72; 2.01)	1.44 (1.33; 1.56)	1.31 (1.19; 1.44)
Christian	2.06 (1.91; 2.23)	1.97 (1.82; 2.13)	1.58 (1.46; 1.72)	1.39 (1.26; 1.53)
Other religion	1.89 (1.72; 2.08)	1.84 (1.66; 2.03)	1.46 (1.32; 1.62)	1.23 (1.10; 1.37)
**SECONDARY OUTCOMES**				
**Recovery**				
Muslim	Referent	Referent	Referent	Referent
No religion	1.97 (1.83; 2.12)	1.89 (1.75; 2.04)	1.40 (1.28; 1.52)	1.28 (1.17; 1.41)
Christian	2.10 (1.95; 2.27)	2.01 (1.86; 2.18)	1.56 (1.43; 1.70)	1.38 (1.25; 1.52)
Other religion	1.96 (1.78; 2.15)	1.91 (1.73; 2.11)	1.47 (1.32; 1.63)	1.24 (1.12; 1.39)
**Reliable improvement**				
Muslim	Referent	Referent	Referent	Referent
No religion	1.47 (1.37; 1.58)	1.40 (1.30; 1.50)	1.44 (1.33; 1.56)	1.29 (1.18; 1.41)
Christian	1.50 (1.40; 1.62)	1.43 (1.33; 1.55)	1.51 (1.39; 1.63)	1.33 (1.21; 1.46)
Other religion	1.36 (1.24; 1.50)	1.30 (1.18; 1.43)	1.35 (1.22; 1.49)	1.17 (1.05; 1.29)
**Reliable deterioration**				
Muslim	Referent	Referent	Referent	Referent
No religion	0.64 (0.57; 0.72)	0.67 (0.59; 0.75)	0.62 (0.55; 0.70)	0.74 (0.64; 0.86)
Christian	0.73 (0.64; 0.82)	0.76 (0.67; 0.85)	0.69 (0.61; 0.78)	0.81 (0.70; 0.94)
Other religion	0.69 (0.59; 0.80)	0.71 (0.61; 0.83)	0.64 (0.55; 0.76)	0.77 (0.65; 0.92)
**Dropout**				
Muslim	Referent	Referent	Referent	Referent
No religion	0.76 (0.70; 0.82)	0.80 (0.73; 0.87)	0.97 (0.88; 1.07)	1.04 (0.93; 1.16)
Christian	0.68 (0.63; 0.74)	0.69 (0.63; 0.76)	0.84 (0.76; 0.92)	0.98 (0.88; 1.10)
Other religion	0.65 (0.59; 0.72)	0.66 (0.59; 0.75)	0.81 (0.71; 0.91)	0.96 (0.84; 1.08)

Model 2: Model 1 additionally adjusted for treatment factors (number of sessions attended, number of sessions cancelled, weeks from referral to assessment, weeks from assessment to first session). Model 3: Model 2 additionally adjusted for clinical factors (PHQ-9 score, GAD-7 score, phobic scale items, diagnosis category, personal functioning (WSAS items)). Model 4: Model 3 additionally adjusted for socio-demographic factors (age, gender, ethnicity, deprivation, long-term health condition, sexual orientation, employment status, medication status). OR=Odds Ratio, CI=Confidence Interval

© 2025 Shafan-Azhar Z et al. *JAMA Network Open*.

First treatment in 2018-2020	Model 1	Model 2	Model 3	Model 4
	Unadjusted	Adjusted for treatment factors	Model 2 additionally adjusted for clinical factors	Model 3 additionally adjusted for socio-demographic factors
	OR (95% CI)	OR (95% CI)	OR (95% CI)	OR (95% CI)
**PRIMARY OUTCOME**				
**Reliable recovery**				
Muslim	Referent	Referent	Referent	Referent
No religion	1.82 (1.66; 1.98)	1.69 (1.54; 1.85)	1.43 (1.30; 1.58)	1.31 (1.17; 1.47)
Christian	1.87 (1.70; 2.05)	1.76 (1.60; 1.94)	1.55 (1.41; 1.72)	1.35 (1.20; 1.52)
Other religion	1.74 (1.56; 1.93)	1.68 (1.50; 1.87)	1.39 (1.24; 1.55)	1.25 (1.11; 1.41)
**SECONDARY OUTCOMES**				
**Recovery**				
Muslim	Referent	Referent	Referent	Referent
No religion	1.91 (1.75; 2.09)	1.78 (1.62; 1.95)	1.45 (1.31; 1.60)	1.33 (1.18; 1.49)
Christian	1.92 (1.75; 2.11)	1.81 (1.65; 1.99)	1.57 (1.42; 1.73)	1.36 (1.21; 1.53)
Other religion	1.81 (1.63; 2.02)	1.76 (1.57; 1.96)	1.40 (1.25; 1.58)	1.26 (1.11; 1.42)
**Reliable improvement**				
Muslim	Referent	Referent	Referent	Referent
No religion	1.44 (1.32; 1.58)	1.36 (1.24; 1.49)	1.49 (1.35; 1.64)	1.41 (1.25; 1.57)
Christian	1.52 (1.38; 1.66)	1.44 (1.31; 1.59)	1.56 (1.41; 1.72)	1.42 (1.26; 1.59)
Other religion	1.44 (1.29; 1.60)	1.38 (1.24; 1.55)	1.51 (1.35; 1.70)	1.40 (1.24; 1.58)
**Reliable deterioration**				
Muslim	Referent	Referent	Referent	Referent
No religion	0.57 (0.49; 0.66)	0.61 (0.52; 0.70)	0.53 (0.46; 0.63)	0.60 (0.50; 0.73)
Christian	0.56 (0.48; 0.66)	0.59 (0.51; 0.70)	0.54 (0.46; 0.63)	0.61 (0.50; 0.74)
Other religion	0.57 (0.47; 0.69)	0.59 (0.49; 0.72)	0.52 (0.43; 0.63)	0.60 (0.48; 0.73)
**Dropout**				
Muslim	Referent	Referent	Referent	Referent
No religion	0.72 (0.66; 0.79)	0.84 (0.75; 0.94)	0.93 (0.83; 1.05)	0.99 (0.86; 1.14)
Christian	0.70 (0.64; 0.77)	0.81 (0.72; 0.91)	0.90 (0.79; 1.01)	1.03 (0.89; 1.19)
Other religion	0.66 (0.59; 0.74)	0.72 (0.62; 0.82)	0.83 (0.72; 0.96)	0.96 (0.83; 1.12)

Model 2: Model 1 additionally adjusted for treatment factors (number of sessions attended, number of sessions cancelled, weeks from referral to assessment, weeks from assessment to first session). Model 3: Model 2 additionally adjusted for clinical factors (PHQ-9 score, GAD-7 score, phobic scale items, diagnosis category, personal functioning (WSAS items)). Model 4: Model 3 additionally adjusted for socio-demographic factors (age, gender, ethnicity, deprivation, long-term health condition, sexual orientation, employment status, medication status). OR=Odds Ratio, CI=Confidence Interval

© 2025 Shafan-Azhar Z et al. *JAMA Network Open*.

eTable 6. Interaction between religion and ethnicity: associations stratified by ethnicity

White	Model 1	Model 2	Model 3	Model 4
	Unadjusted	Adjusted for treatment factors	Model 2 additionally adjusted for clinical factors	Model 3 additionally adjusted for socio-demographic factors
	OR (95% CI)	OR (95% CI)	OR (95% CI)	OR (95% CI)
**PRIMARY OUTCOME**				
**Reliable recovery**				
Muslim	Referent	Referent	Referent	Referent
No religion	2.74 (2.52; 2.99)	2.60 (2.38; 2.84)	1.88 (1.71; 2.06)	1.49 (1.36; 1.64)
Christian	2.74 (2.51; 2.99)	2.59 (2.37; 2.83)	1.94 (1.77; 2.13)	1.50 (1.36; 1.65)
Other religion	2.69 (2.42; 2.99)	2.62 (2.35; 2.91)	1.71 (1.52; 1.91)	1.29 (1.15; 1.45)
**SECONDARY OUTCOMES**				
**Recovery**				
Muslim	Referent	Referent	Referent	Referent
No religion	2.83 (2.60; 3.08)	2.69 (2.47; 2.94)	1.87 (1.70; 2.05)	1.48 (1.35; 1.63)
Christian	2.82 (2.59; 3.08)	2.67 (2.45; 2.92)	1.94 (1.77; 2.13)	1.49 (1.35; 1.65)
Other religion	2.86 (2.58; 3.17)	2.80 (2.52; 3.11)	1.72 (1.53; 1.93)	1.30 (1.15; 1.46)
**Reliable improvement**				
Muslim	Referent	Referent	Referent	Referent
No religion	1.74 (1.62; 1.88)	1.67 (1.55; 1.80)	1.74 (1.60; 1.89)	1.38 (1.27; 1.50)
Christian	1.76 (1.63; 1.90)	1.69 (1.56; 1.82)	1.75 (1.61; 1.90)	1.40 (1.28; 1.52)
Other religion	1.58 (1.44; 1.73)	1.51 (1.37; 1.67)	1.61 (1.45; 1.79)	1.26 (1.13; 1.40)
**Reliable deterioration**				
Muslim	Referent	Referent	Referent	Referent
No religion	0.53 (0.48; 0.60)	0.56 (0.50; 0.63)	0.50 (0.45; 0.57)	0.67 (0.59; 0.76)
Christian	0.56 (0.50; 0.63)	0.59 (0.53; 0.67)	0.54 (0.48; 0.61)	0.71 (0.62; 0.81)
Other religion	0.55 (0.47; 0.64)	0.57 (0.48; 0.66)	0.49 (0.41; 0.57)	0.67 (0.56; 0.79)
**Dropout**				
Muslim	Referent	Referent	Referent	Referent
No religion	1.00 (0.92; 1.09)	0.98 (0.88; 1.08)	1.28 (1.16; 1.42)	1.42 (1.28; 1.59)
Christian	0.88 (0.80; 0.95)	0.86 (0.77; 0.95)	1.11 (1.00; 1.23)	1.37 (1.23; 1.52)
Other religion	0.76 (0.68; 0.84)	0.73 (0.64; 0.83)	1.06 (0.93; 1.21)	1.41 (1.23; 1.61)

Model 2: Model 1 additionally adjusted for treatment factors (number of sessions attended, number of sessions cancelled, weeks from referral to assessment, weeks from assessment to first session). Model 3: Model 2 additionally adjusted for clinical factors (PHQ-9 score, GAD-7 score, phobic scale items, diagnosis category, personal functioning (WSAS items)). Model 4: Model 3 additionally adjusted for socio-demographic factors (age, gender, deprivation, long-term health condition, sexual orientation, employment status, medication status). OR=Odds Ratio, CI=Confidence Interval

© 2025 Shafan-Azhar Z et al. *JAMA Network Open*.

Asian	Model 1	Model 2	Model 3	Model 4
	Unadjusted	Adjusted for treatment factors	Model 2 additionally adjusted for clinical factors	Model 3 additionally adjusted for socio-demographic factors
	OR (95% CI)	OR (95% CI)	OR (95% CI)	OR (95% CI)
**PRIMARY OUTCOME**				
**Reliable recovery**				
Muslim	Referent	Referent	Referent	Referent
No religion	1.31 (1.15; 1.50)	1.27 (1.11; 1.45)	1.17 (1.02; 1.34)	1.11 (0.97; 1.28)
Christian	1.54 (1.28; 1.84)	1.53 (1.28; 1.84)	1.48 (1.23; 1.79)	1.45 (1.20; 1.76)
Other religion	1.54 (1.40; 1.69)	1.49 (1.36; 1.64)	1.35 (1.22; 1.49)	1.26 (1.14; 1.39)
**SECONDARY OUTCOMES**				
**Recovery**				
Muslim	Referent	Referent	Referent	Referent
No religion	1.36 (1.19; 1.54)	1.31 (1.15; 1.49)	1.18 (1.03; 1.36)	1.13 (0.98; 1.30)
Christian	1.53 (1.28; 1.83)	1.52 (1.27; 1.83)	1.46 (1.21; 1.78)	1.44 (1.18; 1.75)
Other religion	1.60 (1.46; 1.75)	1.55 (1.41; 1.70)	1.39 (1.26; 1.54)	1.30 (1.17; 1.44)
**Reliable improvement**				
Muslim	Referent	Referent	Referent	Referent
No religion	1.19 (1.04; 1.35)	1.13 (0.99; 1.29)	1.19 (1.04; 1.37)	1.15 (1.00; 1.32)
Christian	1.30 (1.08; 1.55)	1.29 (1.07; 1.55)	1.38 (1.14; 1.67)	1.34 (1.10; 1.62)
Other religion	1.36 (1.24; 1.50)	1.30 (1.19; 1.43)	1.37 (1.24; 1.51)	1.27 (1.15; 1.41)
**Reliable deterioration**				
Muslim	Referent	Referent	Referent	Referent
No religion	0.79 (0.63; 0.99)	0.83 (0.66; 1.03)	0.76 (0.60; 0.95)	0.81 (0.64; 1.03)
Christian	0.63 (0.45; 0.88)	0.64 (0.46; 0.90)	0.55 (0.39; 0.78)	0.59 (0.41; 0.83)
Other religion	0.72 (0.61; 0.84)	0.75 (0.64; 0.87)	0.67 (0.57; 0.79)	0.73 (0.62; 0.87)
**Dropout**				
Muslim	Referent	Referent	Referent	Referent
No religion	0.70 (0.60; 0.80)	0.76 (0.65; 0.90)	0.79 (0.67; 0.94)	0.79 (0.67; 0.94)
Christian	0.66 (0.55; 0.81)	0.71 (0.57; 0.89)	0.75 (0.60; 0.94)	0.87 (0.69; 1.09)
Other religion	0.64 (0.58; 0.70)	0.68 (0.61; 0.76)	0.73 (0.65; 0.82)	0.82 (0.72; 0.93)

Model 2: Model 1 additionally adjusted for treatment factors (number of sessions attended, number of sessions cancelled, weeks from referral to assessment, weeks from assessment to first session). Model 3: Model 2 additionally adjusted for clinical factors (PHQ-9 score, GAD-7 score, phobic scale items, diagnosis category, personal functioning (WSAS items)). Model 4: Model 3 additionally adjusted for socio-demographic factors (age, gender, deprivation, long-term health condition, sexual orientation, employment status, medication status). OR=Odds Ratio, CI=Confidence Interval

Mixed	Model 1	Model 2	Model 3	Model 4
	Unadjusted	Adjusted for treatment factors	Model 2 additionally adjusted for clinical factors	Model 3 additionally adjusted for socio-demographic factors
	OR (95% CI)	OR (95% CI)	OR (95% CI)	OR (95% CI)
**PRIMARY OUTCOME**				
**Reliable recovery**				
Muslim	Referent	Referent	Referent	Referent
No religion	1.46 (1.15; 1.84)	1.46 (1.15; 1.86)	1.25 (0.97; 1.61)	1.20 (0.92; 1.55)
Christian	1.50 (1.18; 1.90)	1.45 (1.13; 1.85)	1.36 (1.05; 1.76)	1.35 (1.04; 1.76)
Other religion	1.41 (1.03; 1.93)	1.35 (0.97; 1.87)	1.16 (0.82; 1.63)	1.10 (0.78; 1.56)
**SECONDARY OUTCOMES**				
**Recovery**				
Muslim	Referent	Referent	Referent	Referent
No religion	1.41 (1.12; 1.78)	1.41 (1.11; 1.80)	1.17 (0.91; 1.51)	1.13 (0.87; 1.46)
Christian	1.45 (1.15; 1.83)	1.39 (1.09; 1.77)	1.30 (1.00; 1.68)	1.29 (0.99; 1.67)
Other religion	1.34 (0.98; 1.83)	1.28 (0.93; 1.77)	1.07 (0.76; 1.51)	1.02 (0.72; 1.45)
**Reliable improvement**				
Muslim	Referent	Referent	Referent	Referent
No religion	1.33 (1.07; 1.67)	1.31 (1.04; 1.64)	1.35 (1.07; 1.72)	1.27 (1.00; 1.63)
Christian	1.38 (1.10; 1.73)	1.33 (1.05; 1.68)	1.36 (1.07; 1.72)	1.34 (1.05; 1.71)
Other religion	0.94 (0.70; 1.27)	0.87 (0.64; 1.19)	0.89 (0.65; 1.22)	0.84 (0.61; 1.16)
**Reliable deterioration**				
Muslim	Referent	Referent	Referent	Referent
No religion	1.04 (0.70; 1.53)	1.07 (0.72; 1.59)	0.99 (0.66; 1.48)	1.03 (0.69; 1.56)
Christian	0.96 (0.64; 1.43)	1.01 (0.68; 1.51)	0.96 (0.64; 1.44)	0.95 (0.63; 1.44)
Other religion	1.29 (0.78; 2.12)	1.36 (0.82; 2.24)	1.26 (0.75; 2.11)	1.29 (0.76; 2.19)
**Dropout**				
Muslim	Referent	Referent	Referent	Referent
No religion	0.93 (0.74; 1.18)	0.98 (0.73; 1.30)	1.11 (0.82; 1.49)	1.12 (0.83; 1.52)
Christian	0.98 (0.77; 1.25)	1.12 (0.84; 1.50)	1.22 (0.91; 1.64)	1.25 (0.92; 1.69)
Other religion	0.86 (0.62; 1.20)	1.12 (0.75; 1.66)	1.28 (0.86; 1.92)	1.42 (0.94; 2.15)

Model 2: Model 1 additionally adjusted for treatment factors (number of sessions attended, number of sessions cancelled, weeks from referral to assessment, weeks from assessment to first session). Model 3: Model 2 additionally adjusted for clinical factors (PHQ-9 score, GAD-7 score, phobic scale items, diagnosis category, personal functioning (WSAS items)). Model 4: Model 3 additionally adjusted for socio-demographic factors (age, gender, deprivation, long-term health condition, sexual orientation, employment status, medication status). OR=Odds Ratio, CI=Confidence Interval

© 2025 Shafan-Azhar Z et al. *JAMA Network Open*.

Black	Model 1	Model 2	Model 3	Model 4
	Unadjusted	Adjusted for treatment factors	Model 2 additionally adjusted for clinical factors	Model 3 additionally adjusted for socio-demographic factors
	OR (95% CI)	OR (95% CI)	OR (95% CI)	OR (95% CI)
**PRIMARY OUTCOME**				
**Reliable recovery**				
Muslim	Referent	Referent	Referent	Referent
No religion	1.43 (1.18; 1.72)	1.40 (1.15; 1.69)	1.28 (1.05; 1.57)	1.18 (0.96; 1.45)
Christian	1.67 (1.41; 1.98)	1.60 (1.35; 1.90)	1.44 (1.20; 1.72)	1.27 (1.06; 1.53)
Other religion	1.74 (1.31; 2.31)	1.72 (1.29; 2.30)	1.54 (1.14; 2.07)	1.37 (1.01; 1.86)
**SECONDARY OUTCOMES**				
**Recovery**				
Muslim	Referent	Referent	Referent	Referent
No religion	1.45 (1.21; 1.75)	1.43 (1.18; 1.73)	1.27 (1.03; 1.55)	1.17 (0.95; 1.44)
Christian	1.70 (1.44; 2.01)	1.62 (1.37; 1.93)	1.42 (1.18; 1.70)	1.26 (1.05; 1.52)
Other religion	1.68 (1.26; 2.22)	1.66 (1.24; 2.21)	1.42 (1.05; 1.92)	1.27 (0.93; 1.72)
**Reliable improvement**				
Muslim	Referent	Referent	Referent	Referent
No religion	1.24 (1.03; 1.48)	1.17 (0.97; 1.41)	1.28 (1.05; 1.55)	1.19 (0.98; 1.45)
Christian	1.34 (1.14; 1.58)	1.26 (1.07; 1.49)	1.39 (1.17; 1.65)	1.27 (1.06; 1.51)
Other religion	1.38 (1.05; 1.83)	1.32 (0.99; 1.76)	1.50 (1.12; 2.02)	1.36 (1.01; 1.84)
**Reliable deterioration**				
Muslim	Referent	Referent	Referent	Referent
No religion	0.70 (0.53; 0.93)	0.73 (0.55; 0.98)	0.67 (0.49; 0.90)	0.73 (0.54; 0.99)
Christian	0.74 (0.58; 0.95)	0.77 (0.60; 0.99)	0.70 (0.54; 0.91)	0.77 (0.59; 1.00)
Other religion	0.54 (0.33; 0.88)	0.55 (0.34; 0.91)	0.48 (0.29; 0.80)	0.54 (0.32; 0.91)
**Dropout**				
Muslim	Referent	Referent	Referent	Referent
No religion	0.84 (0.69; 1.01)	0.96 (0.77; 1.20)	1.01 (0.81; 1.27)	1.04 (0.83; 1.31)
Christian	0.70 (0.59; 0.83)	0.79 (0.65; 0.96)	0.87 (0.71; 1.07)	0.99 (0.81; 1.22)
Other religion	0.68 (0.51; 0.91)	0.66 (0.47; 0.93)	0.75 (0.53; 1.06)	0.88 (0.62; 1.25)

Model 2: Model 1 additionally adjusted for treatment factors (number of sessions attended, number of sessions cancelled, weeks from referral to assessment, weeks from assessment to first session). Model 3: Model 2 additionally adjusted for clinical factors (PHQ-9 score, GAD-7 score, phobic scale items, diagnosis category, personal functioning (WSAS items)). Model 4: Model 3 additionally adjusted for socio-demographic factors (age, gender, deprivation, long-term health condition, sexual orientation, employment status, medication status). OR=Odds Ratio, CI=Confidence Interval

© 2025 Shafan-Azhar Z et al. *JAMA Network Open*.

Other ethnicity	Model 1	Model 2	Model 3	Model 4
	Unadjusted	Adjusted for treatment factors	Model 2 additionally adjusted for clinical factors	Model 3 additionally adjusted for socio-demographic factors
	OR (95% CI)	OR (95% CI)	OR (95% CI)	OR (95% CI)
**PRIMARY OUTCOME**				
**Reliable recovery**				
Muslim	Referent	Referent	Referent	Referent
No religion	1.66 (1.37; 2.02)	1.67 (1.36; 2.04)	1.43 (1.15; 1.77)	1.22 (0.97; 1.52)
Christian	1.83 (1.47; 2.27)	1.76 (1.41; 2.21)	1.58 (1.24; 2.00)	1.36 (1.06; 1.74)
Other religion	2.11 (1.64; 2.71)	1.99 (1.54; 2.58)	1.69 (1.28; 2.23)	1.49 (1.12; 1.99)
**SECONDARY OUTCOMES**				
**Recovery**				
Muslim	Referent	Referent	Referent	Referent
No religion	1.69 (1.39; 2.05)	1.70 (1.39; 2.07)	1.43 (1.16; 1.78)	1.23 (0.98; 1.54)
Christian	1.76 (1.41; 2.18)	1.70 (1.36; 2.12)	1.51 (1.19; 1.92)	1.29 (1.00; 1.66)
Other religion	2.20 (1.71; 2.83)	2.09 (1.61; 2.71)	1.78 (1.35; 2.37)	1.55 (1.16; 2.08)
**Reliable improvement**				
Muslim	Referent	Referent	Referent	Referent
No religion	1.31 (1.09; 1.58)	1.31 (1.09; 1.58)	1.34 (1.10; 1.63)	1.10 (0.90; 1.35)
Christian	1.37 (1.12; 1.69)	1.33 (1.08; 1.65)	1.31 (1.05; 1.63)	1.10 (0.87; 1.39)
Other religion	1.67 (1.30; 2.15)	1.59 (1.23; 2.05)	1.54 (1.18; 2.00)	1.32 (1.00; 1.73)
**Reliable deterioration**				
Muslim	Referent	Referent	Referent	Referent
No religion	0.68 (0.51; 0.90)	0.68 (0.51; 0.91)	0.60 (0.44; 0.82)	0.78 (0.57; 1.09)
Christian	0.68 (0.49; 0.94)	0.70 (0.50; 0.97)	0.67 (0.47; 0.95)	0.86 (0.60; 1.25)
Other religion	0.59 (0.39; 0.89)	0.62 (0.41; 0.94)	0.62 (0.40; 0.95)	0.81 (0.51; 1.27)
**Dropout**				
Muslim	Referent	Referent	Referent	Referent
No religion	0.95 (0.77; 1.16)	0.95 (0.75; 1.21)	1.06 (0.83; 1.35)	1.13 (0.88; 1.46)
Christian	0.92 (0.73; 1.15)	1.02 (0.78; 1.34)	1.16 (0.88; 1.54)	1.35 (1.01; 1.81)
Other religion	0.76 (0.58; 1.00)	0.79 (0.57; 1.09)	0.88 (0.63; 1.22)	1.00 (0.71; 1.42)

Model 2: Model 1 additionally adjusted for treatment factors (number of sessions attended, number of sessions cancelled, weeks from referral to assessment, weeks from assessment to first session). Model 3: Model 2 additionally adjusted for clinical factors (PHQ-9 score, GAD-7 score, phobic scale items, diagnosis category, personal functioning (WSAS items)). Model 4: Model 3 additionally adjusted for socio-demographic factors (age, gender, deprivation, long-term health condition, sexual orientation, employment status, medication status). OR=Odds Ratio, CI=Confidence Interval

© 2025 Shafan-Azhar Z et al. *JAMA Network Open*.

eTable 7. Participant characteristics of Muslim Patients of each ethnic group

Characteristics of Muslim participants at initial assessment					
**Ethnicity**	**White**	**Asian**	**Mixed**	**Black**	**Other**
	N=3,280	N=4,876	N=403	N=705	N=971
**DEMOGRAPHICS**					
Age at referral - Mean (SD)	40.3 (11.8)	36.0 (11.8)	35.9 (11.7)	35.4 (11.8)	39.2 (12.4)
	**N (%)**	**N (%)**	**N (%)**	**N (%)**	**N (%)**
**Gender**					
Female	2,468 (75.2)	3,054 (62.6)	278 (69.0)	494 (70.1)	658 (67.8)
Male	809 (24.7)	1,821 (37.3)	125 (31.0)	210 (29.8)	310 (31.9)
Missing	3 (0.1)	1 (0.0)	0 (0.0)	1 (0.1)	3 (0.3)
**IMD decile**					
1 (Most deprived)	749 (22.8)	202 (4.1)	59 (14.6)	130 (18.4)	153 (15.8)
2	982 (29.9)	992 (20.3)	86 (21.3)	234 (33.2)	264 (27.2)
3	665 (20.3)	977 (20.0)	96 (23.8)	144 (20.4)	188 (19.4)
4	329 (10.0)	890 (18.3)	48 (11.9)	75 (10.6)	113 (11.6)
5	188 (5.7)	676 (13.9)	41 (10.2)	46 (6.5)	89 (9.2)
6	152 (4.6)	527 (10.8)	32 (7.9)	32 (4.5)	69 (7.1)
7	76 (2.3)	256 (5.3)	23 (5.7)	16 (2.3)	46 (4.7)
8	83 (2.5)	176 (3.6)	11 (2.7)	13 (1.8)	23 (2.4)
9	22 (0.7)	90 (1.8)	2 (0.5)	6 (0.9)	15 (1.5)
10 (Least deprived)	14 (0.4)	35 (0.7)	2 (0.5)	2 (0.3)	7 (0.7)
Missing	20 (0.6)	55 (1.1)	3 (0.7)	7 (1.0)	4 (0.4)
**Employment status**					
Employed	744 (22.7)	2,169 (44.5)	176 (43.7)	257 (36.5)	267 (27.5)
Unemployed	129 (3.9)	253 (5.2)	27 (6.7)	43 (6.1)	63 (6.5)
Student	167 (5.1)	475 (9.7)	35 (8.7)	97 (13.8)	86 (8.9)
Long-term sick	1,001 (30.5)	465 (9.5)	60 (14.9)	128 (18.2)	241 (24.8)
Homemaker	481 (14.7)	531 (10.9)	38 (9.4)	42 (6.0)	119 (12.3)
Not seeking work	657 (20.0)	815 (16.7)	57 (14.1)	124 (17.6)	153 (15.8)
Volunteer	12 (0.4)	23 (0.5)	2 (0.5)	3 (0.4)	13 (1.3)
Retired	63 (1.9)	112 (2.3)	6 (1.5)	7 (1.0)	16 (1.6)
Missing	26 (0.8)	33 (0.7)	2 (0.5)	4 (0.6)	13 (1.3)
**Sexual orientation**					
Heterosexual	3,135 (95.6)	4,645 (95.3)	383 (95.0)	655 (92.9)	902 (92.9)
Gay/Lesbian	16 (0.5)	56 (1.1)	4 (1.0)	7 (1.0)	7 (0.7)
Bisexual	6 (0.2)	18 (0.4)	2 (0.5)	4 (0.6)	5 (0.5)
Missing	123 (3.8)	157 (3.2)	14 (3.5)	39 (5.5)	57 (5.9)
**CLINICAL MEASURES PRE-TREATMENT, PRE-EXISTING CONDITIONS AND MEDICATION**					
	**Mean (SD)**	**Mean (SD)**	**Mean (SD)**	**Mean (SD)**	**Mean (SD)**
Depression symptoms pre-treatment (PHQ-9)	17.7 (5.9)	16.6 (5.9)	16.7 (6.0)	16.9 (5.8)	16.9 (5.8)
Anxiety symptoms pre-treatment (GAD-7)	15.3 (4.7)	14.9 (4.8)	14.5 (4.7)	14.6 (5.0)	15.1 (4.6)

© 2025 Shafan-Azhar Z et al. *JAMA Network Open*.

WSAS – home	4.5 (2.6)	4.2 (2.6)	4.2 (2.5)	4.1 (2.7)	4.4 (2.6)
WSAS – social	5.0 (2.6)	4.8 (2.6)	5.0 (2.6)	5.1 (2.7)	4.8 (2.6)
WSAS – relationships	4.7 (2.7)	4.4 (2.7)	4.4 (2.7)	4.5 (2.8)	4.4 (2.8)
WSAS – leisure	4.3 (2.6)	4.4 (2.6)	4.5 (2.5)	4.4 (2.7)	4.0 (2.8)
	**N (%)**	**N (%)**	**N (%)**	**N (%)**	**N (%)**
**Psychotropic medication**					
Not prescribed	1,187 (36.2)	2,507 (51.4)	208 (51.6)	375 (53.2)	402 (41.4)
Prescribed and taking	1,709 (52.1)	1,755 (36.0)	147 (36.5)	244 (34.6)	458 (47.2)
Prescribed not taking	155 (4.7)	212 (4.3)	26 (6.5)	21 (3.0)	52 (5.4)
Missing	229 (7.0)	402 (8.2)	22 (5.5)	65 (9.2)	59 (6.1)
**Self-reported long-term condition**					
No	1,515 (46.2)	3,092 (63.4)	244 (60.5)	424 (60.1)	485 (49.9)
Yes	1,056 (32.2)	1,401 (28.7)	90 (22.3)	184 (26.1)	305 (31.4)
Missing	709 (21.6)	383 (7.9)	69 (17.1)	97 (13.8)	181 (18.6)
**TREATMENT FACTORS**					
	**N (%)**	**N (%)**	**N (%)**	**N (%)**	**N (%)**
**Diagnosis category**					
Depression	1,466 (44.7)	2,950 (60.5)	197 (48.9)	332 (47.1)	430 (44.3)
GAD	337 (10.3)	381 (7.8)	54 (13.4)	70 (9.9)	129 (13.3)
Mixed anxiety and depression	344 (10.5)	295 (6.1)	31 (7.7)	62 (8.8)	85 (8.8)
OCD	64 (2.0)	83 (1.7)	8 (2.0)	2 (0.3)	21 (2.2)
PTSD	296 (9.0)	227 (4.7)	25 (6.2)	59 (8.4)	76 (7.8)
Phobia and panic	281 (8.6)	304 (6.2)	33 (8.2)	74 (10.5)	90 (9.3)
Unspecified anxiety disorder	35 (1.1)	295 (6.1)	10 (2.5)	18 (2.6)	10 (1.0)
Other	56 (1.7)	36 (0.7)	4 (1.0)	12 (1.7)	18 (1.9)
Missing	401 (12.2)	305 (6.3)	41 (10.2)	76 (10.8)	112 (11.5)
	**Mean (SD)**	**Mean (SD)**	**Mean (SD)**	**Mean (SD)**	**Mean (SD)**
Number of sessions	7.5 (4.6)	7.1 (4.6)	7.6 (5.8)	6.6 (4.4)	7.1 (4.2)
Number of cancelled sessions	1.5 (1.7)	1.5 (1.7)	1.6 (1.7)	1.4 (1.6)	1.5 (1.7)
Time between referral and assessment (weeks)	6.8 (13.3)	3.1 (6.2)	4.1 (5.8)	4.0 (8.3)	4.7 (7.2)
Time between assessment and treatment (weeks)	11.2 (10.9)	10.6 (8.6)	9.9 (9.6)	10.1 (9.8)	12.3 (11.0)

Abbreviations: IMD = Index of multiple deprivation, GAD = Generalized anxiety disorder, OCD=Obsessive compulsive disorder, PTSD=Post traumatic stress disorder, PHQ=Patient health questionnaire, SD=Standard Deviation, LTC=Long term condition

© 2025 Shafan-Azhar Z et al. *JAMA Network Open*.

eTable 8. Complete case analysis results

Complete case analysis	Model 1	Model 2	Model 3	Model 4
	Unadjusted	Adjusted for treatment factors	Model 2 additionally adjusted for clinical factors	Model 3 additionally adjusted for socio-demographic factors
	OR (95% CI)	OR (95% CI)	OR (95% CI)	OR (95% CI)
**PRIMARY OUTCOME**				
**Reliable recovery**				
Muslim	Referent	Referent	Referent	Referent
No religion	1.91 (1.81; 2.00)	1.83 (1.74; 1.93)	1.47 (1.39; 1.55)	1.42 (1.31; 1.53)
Christian	1.94 (1.84; 2.04)	1.85 (1.76; 1.95)	1.55 (1.47; 1.65)	1.45 (1.34; 1.57)
Other religion	1.91 (1.79; 2.03)	1.85 (1.73; 1.97)	1.46 (1.36; 1.57)	1.29 (1.18; 1.40)
**SECONDARY OUTCOMES**				
**Recovery**				
Muslim	Referent	Referent	Referent	Referent
No religion	1.95 (1.86; 2.05)	1.88 (1.79; 1.98)	1.46 (1.38; 1.55)	1.42 (1.31; 1.53)
Christian	1.97 (1.88; 2.07)	1.89 (1.79; 1.99)	1.55 (1.46; 1.64)	1.44 (1.33; 1.56)
Other religion	1.99 (1.87; 2.12)	1.94 (1.82; 2.07)	1.49 (1.38; 1.60)	1.30 (1.19; 1.41)
**Reliable improvement**				
Muslim	Referent	Referent	Referent	Referent
No religion	1.43 (1.37; 1.50)	1.37 (1.30; 1.44)	1.46 (1.38; 1.55)	1.37 (1.27; 1.47)
Christian	1.47 (1.40; 1.54)	1.41 (1.34; 1.48)	1.52 (1.44; 1.61)	1.39 (1.29; 1.50)
Other religion	1.41 (1.33; 1.50)	1.33 (1.25; 1.42)	1.44 (1.34; 1.55)	1.27 (1.17; 1.38)
**Reliable deterioration**				
Muslim	Referent	Referent	Referent	Referent
No religion	0.64 (0.59; 0.69)	0.65 (0.60; 0.70)	0.57 (0.52; 0.63)	0.67 (0.59; 0.75)
Christian	0.68 (0.63; 0.73)	0.69 (0.63; 0.74)	0.62 (0.57; 0.68)	0.71 (0.63; 0.80)
Other religion	0.64 (0.58; 0.70)	0.64 (0.57; 0.71)	0.57 (0.51; 0.64)	0.70 (0.61; 0.80)
**Dropout**				
Muslim	Referent	Referent	Referent	Referent
No religion	0.83 (0.79; 0.87)	0.88 (0.83; 0.93)	1.04 (0.97; 1.12)	1.05 (0.96; 1.15)
Christian	0.76 (0.72; 0.80)	0.80 (0.75; 0.85)	0.94 (0.88; 1.00)	1.07 (0.97; 1.17)
Other religion	0.67 (0.63; 0.72)	0.70 (0.65; 0.76)	0.87 (0.80; 0.95)	1.02 (0.92; 1.13)

Model 2: Model 1 additionally adjusted for treatment factors (number of sessions attended, number of sessions cancelled, weeks from referral to assessment, weeks from assessment to first session). Model 3: Model 2 additionally adjusted for clinical factors (PHQ-9 score, GAD-7 score, phobic scale items, diagnosis category, personal functioning (WSAS items)). Model 4: Model 3 additionally adjusted for socio-demographic factors (age, gender, ethnicity, deprivation, long-term health condition, sexual orientation, employment status, medication status). OR=Odds Ratio, CI=Confidence Interval

© 2025 Shafan-Azhar Z et al. *JAMA Network Open*.

eTable 9. Sensitivity analyses with more granular other religion categories

Participant characteristics for further subgroups within the 'Other religion (N=8,405)' category in the main analysis					
	**Jewish** N=2,234	**Hindu** N=2,152	**Sikh** N=1,166	**Buddhist** N=407	**Other** N=2,446
**DEMOGRAPHICS**					
Age at referral - Mean (SD)	47.2 (17.7)	40.2 (14.1)	39.9 (13.4)	41.9 (14.1)	41.3 (14.8)
	**N (%)**	**N (%)**	**N (%)**	**N (%)**	**N (%)**
**Ethnicity**					
White	2,115 (94.7)	27 (1.3)	6 (0.5)	148 (36.4)	1,613 (65.9)
Asian	8 (0.4)	1,957 (90.9)	1,123 (96.3)	118 (29.0)	208 (8.5)
Mixed	47 (2.1)	61 (2.8)	24 (2.1)	26 (6.4)	163 (6.7)
Black	7 (0.3)	28 (1.3)	1 (0.1)	32 (7.9)	242 (9.9)
Other	47 (2.1)	70 (3.3)	7 (0.6)	75 (18.4)	184 (7.5)
Missing	10 (0.4)	9 (0.4)	5 (0.4)	8 (2.0)	36 (1.5)
**Gender**					
Female	1,493 (66.8)	1,405 (65.3)	791 (67.8)	271 (66.6)	1,671 (68.3)
Male	735 (32.9)	745 (34.6)	373 (32.0)	136 (33.4)	773 (31.6)
Missing	6 (0.3)	2 (0.1)	2 (0.2)	0 (0.0)	2 (0.1)
**IMD decile**					
1 (Most deprived)	20 (0.9)	74 (3.4)	17 (1.5)	33 (8.1)	255 (10.4)
2	75 (3.4)	225 (10.5)	144 (12.3)	68 (16.7)	494 (20.2)
3	138 (6.2)	332 (15.4)	179 (15.4)	61 (15.0)	426 (17.4)
4	194 (8.7)	365 (17.0)	253 (21.7)	62 (15.2)	324 (13.2)
5	289 (12.9)	335 (15.6)	195 (16.7)	47 (11.5)	236 (9.6)
6	427 (19.1)	323 (15.0)	146 (12.5)	44 (10.8)	263 (10.8)
7	409 (18.3)	188 (8.7)	85 (7.3)	40 (9.8)	170 (7.0)
8	314 (14.1)	166 (7.7)	81 (6.9)	33 (8.1)	153 (6.3)
9	200 (9.0)	76 (3.5)	36 (3.1)	9 (2.2)	68 (2.8)
10 (Least deprived)	102 (4.6)	42 (2.0)	9 (0.8)	2 (0.5)	24 (1.0)
Missing	66 (3.0)	26 (1.2)	21 (1.8)	8 (2.0)	33 (1.3)
**Employment status**					
Employed	1,270 (56.8)	1,277 (59.3)	655 (56.2)	214 (52.6)	1,252 (51.2)
Unemployed	102 (4.6)	127 (5.9)	66 (5.7)	27 (6.6)	136 (5.6)
Student	104 (4.7)	154 (7.2)	73 (6.3)	25 (6.1)	146 (6.0)
Long-term sick	108 (4.8)	94 (4.4)	80 (6.9)	35 (8.6)	308 (12.6)
Homemaker	96 (4.3)	100 (4.6)	67 (5.7)	16 (3.9)	113 (4.6)
Not seeking work	128 (5.7)	217 (10.1)	159 (13.6)	55 (13.5)	259 (10.6)
Volunteer	20 (0.9)	14 (0.7)	8 (0.7)	5 (1.2)	20 (0.8)
Retired	381 (17.1)	154 (7.2)	55 (4.7)	29 (7.1)	189 (7.7)
Missing	25 (1.1)	15 (0.7)	3 (0.3)	1 (0.2)	23 (0.9)
**Sexual orientation**					
Heterosexual	2,088 (93.5)	2,043 (94.9)	1,109 (95.1)	359 (88.2)	2,200 (89.9)
Gay/Lesbian	35 (1.6)	19 (0.9)	3 (0.3)	11 (2.7)	67 (2.7)
Bisexual	29 (1.3)	11 (0.5)	7 (0.6)	17 (4.2)	61 (2.5)
Missing	82 (3.7)	79 (3.7)	47 (4.0)	20 (4.9)	118 (4.8)
**CLINICAL MEASURES PRE-TREATMENT, PRE-EXISTING CONDITIONS AND MEDICATION**					
	**Mean (SD)**	**Mean (SD)**	**Mean (SD)**	**Mean (SD)**	**Mean (SD)**
Depression symptoms pre-treatment (PHQ-9)	12.4 (6.1)	15.0 (6.2)	16.1 (5.9)	15.6 (6.3)	15.2 (6.2)
Anxiety symptoms pre-treatment (GAD-7)	12.0 (5.2)	13.8 (5.0)	14.4 (4.9)	13.7 (5.1)	13.6 (5.1)
WSAS – home	3.0 (2.4)	3.7 (2.6)	3.7 (2.6)	3.7 (2.5)	3.8 (2.5)
WSAS – social	3.5 (2.5)	4.3 (2.6)	4.5 (2.5)	4.4 (2.5)	4.5 (2.6)
WSAS – relationships	2.8 (2.5)	3.8 (2.7)	4.1 (2.6)	3.9 (2.7)	3.8 (2.7)

© 2025 Shafan-Azhar Z et al. *JAMA Network Open*.

WSAS – leisure	3.3 (2.5)	4.2 (2.6)	4.4 (2.6)	4.3 (2.5)	4.1 (2.6)
	**N (%)**	**N (%)**	**N (%)**	**N (%)**	**N (%)**
**Psychotropic medication**					
Not prescribed	1,229 (55.0)	1,273 (59.2)	671 (57.5)	230 (56.5)	1,305 (53.4)
Prescribed and taking	769 (34.4)	604 (28.1)	352 (30.2)	130 (31.9)	884 (36.1)
Prescribed not taking	94 (4.2)	102 (4.7)	60 (5.1)	16 (3.9)	107 (4.4)
Missing	142 (6.4)	173 (8.0)	83 (7.1)	31 (7.6)	150 (6.1)
**Self-reported long-term condition**					
No	1,197 (53.6)	1,251 (58.1)	719 (61.7)	216 (53.1)	1,294 (52.9)
Yes	719 (32.2)	666 (30.9)	362 (31.0)	130 (31.9)	845 (34.5)
Missing	318 (14.2)	235 (10.9)	85 (7.3)	61 (15.0)	307 (12.6)
**TREATMENT FACTORS**					
	**N (%)**	**N (%)**	**N (%)**	**N (%)**	**N (%)**
**Diagnosis category**					
Depression	1,090 (48.8)	1,293 (60.1)	836 (71.7)	213 (52.3)	1,146 (46.9)
GAD	427 (19.1)	224 (10.4)	66 (5.7)	54 (13.3)	386 (15.8)
Mixed anxiety and depression	126 (5.6)	129 (6.0)	65 (5.6)	34 (8.4)	166 (6.8)
OCD	50 (2.2)	32 (1.5)	17 (1.5)	2 (0.5)	43 (1.8)
PTSD	31 (1.4)	56 (2.6)	21 (1.8)	16 (3.9)	101 (4.1)
Phobia and panic	127 (5.7)	119 (5.5)	42 (3.6)	19 (4.7)	194 (7.9)
Anxiety disorder not otherwise specified	58 (2.6)	76 (3.5)	64 (5.5)	10 (2.5)	42 (1.7)
Other	17 (0.8)	25 (1.2)	8 (0.7)	4 (1.0)	46 (1.9)
Missing	308 (13.8)	198 (9.2)	47 (4.0)	55 (13.5)	322 (13.2)
	**Mean (SD)**	**Mean (SD)**	**Mean (SD)**	**Mean (SD)**	**Mean (SD)**
Number of sessions	7.7 (4.7)	7.6 (4.6)	7.7 (5.1)	8.3 (4.8)	7.8 (4.8)
Number of cancelled sessions	1.5 (1.7)	1.5 (1.7)	1.7 (1.9)	1.5 (1.6)	1.5 (1.7)
Time between referral and assessment (weeks)	5.0 (7.3)	3.6 (6.5)	2.6 (4.4)	3.4 (5.4)	4.1 (8.1)
Time between assessment and treatment (weeks)	11.6 (9.7)	10.6 (8.7)	11.0 (8.2)	10.0 (9.4)	10.0 (9.5)

One-way ANOVA was used for continuous variables and chi-square tests were used for categorical variables to test for differences in patient characteristics across the religion categories; all p-values < 0.001 so not presented in the table above. Abbreviations: IMD = Index of multiple deprivation, GAD = Generalized anxiety disorder, OCD=Obsessive compulsive disorder, PTSD=Post traumatic stress disorder, PHQ=Patient health questionnaire, SD=Standard Deviation, LTC=Long term condition

© 2025 Shafan-Azhar Z et al. *JAMA Network Open*.

Multiple imputation (main analysis)	Model 1 (unadjusted)	Model 2	Model 3	Model 4
**PRIMARY OUTCOME**	OR (95% CI)	OR (95% CI)	OR (95% CI)	OR (95% CI)
**Reliable recovery**				
Muslim	Referent	Referent	Referent	Referent
Jewish	2.11 (1.91; 2.33)	2.08 (1.88; 2.30)	1.29 (1.16; 1.44)	1.08 (0.97; 1.22)
Hindu	2.01 (1.82; 2.22)	1.94 (1.75; 2.14)	1.61 (1.45; 1.79)	1.32 (1.18; 1.47)
Sikh	1.62 (1.43; 1.84)	1.57 (1.38; 1.79)	1.40 (1.22; 1.60)	1.16 (1.00; 1.33)
Buddhist	1.84 (1.50; 2.27)	1.66 (1.35; 2.06)	1.43 (1.15; 1.79)	1.28 (1.03; 1.61)
Other religion	1.81 (1.65; 1.99)	1.75 (1.59; 1.92)	1.47 (1.33; 1.63)	1.37 (1.23; 1.53)
**SECONDARY OUTCOMES**				
**Recovery**				
Muslim	Referent	Referent	Referent	Referent
Jewish	2.30 (2.08; 2.54)	2.27 (2.05; 2.51)	1.31 (1.18; 1.46)	1.10 (0.98; 1.24)
Hindu	2.13 (1.93; 2.35)	2.06 (1.86; 2.27)	1.68 (1.51; 1.87)	1.36 (1.22; 1.52)
Sikh	1.63 (1.43; 1.85)	1.58 (1.39; 1.80)	1.40 (1.22; 1.60)	1.15 (1.00; 1.33)
Buddhist	1.82 (1.48; 2.24)	1.64 (1.33; 2.03)	1.39 (1.11; 1.74)	1.25 (0.99; 1.56)
Other religion	1.86 (1.69; 2.04)	1.80 (1.63; 1.97)	1.47 (1.33; 1.63)	1.38 (1.24; 1.53)
**Reliable improvement**				
Muslim	Referent	Referent	Referent	Referent
Jewish	1.29 (1.17; 1.42)	1.24 (1.12; 1.36)	1.33 (1.20; 1.47)	1.14 (1.02; 1.27)
Hindu	1.54 (1.39; 1.70)	1.47 (1.33; 1.63)	1.55 (1.40; 1.72)	1.30 (1.17; 1.45)
Sikh	1.49 (1.31; 1.70)	1.42 (1.25; 1.62)	1.43 (1.25; 1.64)	1.24 (1.07; 1.42)
Buddhist	1.48 (1.20; 1.82)	1.32 (1.06; 1.63)	1.38 (1.11; 1.72)	1.24 (0.99; 1.55)
Other religion	1.38 (1.26; 1.51)	1.31 (1.19; 1.44)	1.39 (1.26; 1.53)	1.29 (1.17; 1.43)
**Reliable deterioration**				
Muslim	Referent	Referent	Referent	Referent
Jewish	0.60 (0.51; 0.71)	0.62 (0.52; 0.73)	0.51 (0.43; 0.61)	0.67 (0.55; 0.81)
Hindu	0.56 (0.47; 0.67)	0.58 (0.48; 0.69)	0.53 (0.44; 0.63)	0.66 (0.54; 0.79)
Sikh	0.76 (0.61; 0.93)	0.78 (0.63; 0.96)	0.76 (0.61; 0.94)	0.92 (0.73; 1.15)
Buddhist	0.74 (0.52; 1.05)	0.81 (0.57; 1.14)	0.74 (0.52; 1.05)	0.84 (0.59; 1.21)
Other religion	0.66 (0.56; 0.77)	0.69 (0.59; 0.81)	0.62 (0.52; 0.73)	0.68 (0.58; 0.81)
**Dropout**				
Muslim	Referent	Referent	Referent	Referent
Jewish	0.51 (0.46; 0.57)	0.53 (0.47; 0.60)	0.78 (0.68; 0.89)	1.09 (0.95; 1.26)
Hindu	0.69 (0.62; 0.77)	0.71 (0.63; 0.81)	0.83 (0.73; 0.94)	0.95 (0.83; 1.08)
Sikh	0.90 (0.78; 1.03)	0.88 (0.75; 1.03)	0.95 (0.81; 1.12)	1.06 (0.90; 1.26)
Buddhist	0.54 (0.42; 0.69)	0.66 (0.50; 0.88)	0.75 (0.56; 1.00)	0.89 (0.67; 1.19)
Other religion	0.74 (0.67; 0.82)	0.80 (0.71; 0.90)	0.93 (0.83; 1.05)	1.08 (0.96; 1.23)

Model 2: Model 1 additionally adjusted for treatment factors (number of sessions attended, number of sessions cancelled, weeks from referral to assessment, weeks from assessment to first session). Model 3: Model 2 additionally adjusted for clinical factors (PHQ-9 score, GAD-7 score, phobic scale items, diagnosis category, personal functioning (WSAS items)). Model 4: Model 3 additionally adjusted for socio-demographic factors (age, gender, ethnicity, deprivation, long-term health condition, sexual orientation, employment status, medication status). OR=Odds Ratio, CI=Confidence Interval

© 2025 Shafan-Azhar Z et al. *JAMA Network Open*.

Complete case (sensitivity analysis)	Model 1 (unadjusted)	Model 2	Model 3	Model 4
**PRIMARY OUTCOME**	OR (95% CI)	OR (95% CI)	OR (95% CI)	OR (95% CI)
**Reliable recovery**				
Muslim	Referent	Referent	Referent	Referent
Jewish	2.11 (1.91; 2.33)	2.10 (1.89; 2.33)	1.29 (1.15; 1.45)	1.11 (0.96; 1.29)
Hindu	2.01 (1.82; 2.22)	1.95 (1.76; 2.16)	1.66 (1.48; 1.85)	1.30 (1.14; 1.48)
Sikh	1.62 (1.43; 1.84)	1.59 (1.39; 1.82)	1.37 (1.18; 1.58)	1.22 (1.03; 1.44)
Buddhist	1.84 (1.50; 2.27)	1.64 (1.31; 2.04)	1.42 (1.11; 1.82)	1.45 (1.09; 1.94)
Other religion	1.81 (1.65; 1.99)	1.74 (1.58; 1.92)	1.48 (1.33; 1.66)	1.43 (1.25; 1.64)
**SECONDARY OUTCOMES**				
**Recovery**				
Muslim	Referent	Referent	Referent	Referent
Jewish	2.30 (2.08; 2.54)	2.32 (2.08; 2.58)	1.34 (1.19; 1.51)	1.14 (0.98; 1.33)
Hindu	2.13 (1.93; 2.35)	2.07 (1.87; 2.30)	1.74 (1.55; 1.95)	1.34 (1.17; 1.53)
Sikh	1.63 (1.43; 1.85)	1.60 (1.40; 1.83)	1.37 (1.19; 1.58)	1.21 (1.03; 1.43)
Buddhist	1.82 (1.48; 2.24)	1.62 (1.30; 2.02)	1.40 (1.09; 1.80)	1.43 (1.06; 1.92)
Other religion	1.86 (1.69; 2.04)	1.79 (1.62; 1.97)	1.48 (1.32; 1.65)	1.42 (1.24; 1.62)
**Reliable improvement**				
Muslim	Referent	Referent	Referent	Referent
Jewish	1.29 (1.17; 1.42)	1.21 (1.09; 1.34)	1.36 (1.21; 1.52)	1.18 (1.02; 1.36)
Hindu	1.54 (1.39; 1.70)	1.47 (1.32; 1.63)	1.55 (1.39; 1.74)	1.32 (1.15; 1.51)
Sikh	1.49 (1.31; 1.70)	1.43 (1.25; 1.64)	1.42 (1.23; 1.64)	1.23 (1.04; 1.45)
Buddhist	1.48 (1.20; 1.82)	1.32 (1.05; 1.64)	1.47 (1.14; 1.89)	1.47 (1.09; 1.99)
Other religion	1.38 (1.26; 1.51)	1.30 (1.17; 1.43)	1.43 (1.28; 1.60)	1.31 (1.15; 1.49)
**Reliable deterioration**				
Muslim	Referent	Referent	Referent	Referent
Jewish	0.60 (0.51; 0.71)	0.60 (0.50; 0.72)	0.49 (0.40; 0.60)	0.73 (0.57; 0.94)
Hindu	0.56 (0.47; 0.67)	0.55 (0.46; 0.67)	0.49 (0.40; 0.60)	0.57 (0.45; 0.73)
Sikh	0.76 (0.61; 0.93)	0.73 (0.58; 0.91)	0.70 (0.56; 0.89)	0.83 (0.63; 1.09)
Buddhist	0.74 (0.52; 1.05)	0.86 (0.60; 1.22)	0.75 (0.51; 1.12)	0.80 (0.50; 1.30)
Other religion	0.66 (0.56; 0.77)	0.68 (0.57; 0.80)	0.63 (0.52; 0.76)	0.71 (0.57; 0.88)
**Dropout**				
Muslim	Referent	Referent	Referent	Referent
Jewish	0.51 (0.46; 0.57)	0.52 (0.45; 0.59)	0.74 (0.64; 0.86)	1.04 (0.87; 1.25)
Hindu	0.69 (0.62; 0.77)	0.72 (0.63; 0.81)	0.85 (0.74; 0.97)	0.92 (0.79; 1.08)
Sikh	0.90 (0.78; 1.03)	0.87 (0.74; 1.03)	0.95 (0.80; 1.13)	1.02 (0.84; 1.25)
Buddhist	0.54 (0.42; 0.69)	0.66 (0.49; 0.88)	0.78 (0.56; 1.07)	0.87 (0.60; 1.26)
Other religion	0.74 (0.67; 0.82)	0.81 (0.71; 0.91)	0.98 (0.86; 1.11)	1.13 (0.97; 1.32)

Model 2: Model 1 additionally adjusted for treatment factors (number of sessions attended, number of sessions cancelled, weeks from referral to assessment, weeks from assessment to first session). Model 3: Model 2 additionally adjusted for clinical factors (PHQ-9 score, GAD-7 score, phobic scale items, diagnosis category, personal functioning (WSAS items)). Model 4: Model 3 additionally adjusted for socio-demographic factors (age, gender, ethnicity, deprivation, long-term health condition, sexual orientation, employment status, medication status). OR=Odds Ratio, CI=Confidence Interval

© 2025 Shafan-Azhar Z et al. *JAMA Network Open*.

eReferences

1. Chambless DL, Caputo GC, Jasin SE, Gracely EJ, Williams C. The Mobility Inventory for Agoraphobia. *Behaviour Research and Therapy*. 1985/01/01/ 1985;23(1):35-44. doi:https://doi.org/10.1016/0005-7967(85)90140-8
2. Salkovskis PM, Rimes KA, Warwick HMC, Clark DM. The Health Anxiety Inventory: development and validation of scales for the measurement of health anxiety and hypochondriasis. *Psychological Medicine*. 2002;32(5):843-853. doi:10.1017/S0033291702005822
3. Foa EB, Kozak MJ, Salkovskis PM, Coles ME, Amir N. The validation of a new obsessive–compulsive disorder scale: The Obsessive–Compulsive Inventory. *Psychological Assessment*. 1998;10:206-214. doi:10.1037/1040-3590.10.3.206
4. Shear MK, Rucci P, Williams J, et al. Reliability and validity of the Panic Disorder Severity Scale: replication and extension. *Journal of Psychiatric Research*. 2001/09/01/ 2001;35(5):293-296. doi:https://doi.org/10.1016/S0022-3956(01)00028-0
5. Creamer M, Bell R, Failla S. Psychometric properties of the Impact of Event Scale—Revised. *Behaviour Research and Therapy*. 2003/12/01/ 2003;41(12):1489-1496. doi:https://doi.org/10.1016/j.brat.2003.07.010
6. Geier TJ, Hunt JC, Hanson JL, et al. Validation of Abbreviated Four- and Eight-Item Versions of the PTSD Checklist for DSM-5 in a Traumatically Injured Sample. *J Trauma Stress*. Jun 2020;33(3):218-226. doi:10.1002/jts.22478
7. Connor KM, Davidson JRT, Churchill LE, Sherwood A, Weisler RH, Foa E. Psychometric properties of the Social Phobia Inventory (SPIN): New self-rating scale. *The British Journal of Psychiatry*. 2000;176(4):379-386. doi:10.1192/bjp.176.4.379

© 2025 Shafan-Azhar Z et al. *JAMA Network Open*.
