# Supplementary material for: Psychological Therapy Outcomes and Engagement in People of Different Religions
Source: JAMA Netw Open. 2025 Apr 8;8(4):e254026. doi: 10.1001/jamanetworkopen.2025.4026 (PMC11979733; doi:10.1001/jamanetworkopen.2025.4026)
Supplement: Supplement 2. — Data Sharing Statement [file jamanetwopen-e254026-s002.pdf]

Data Sharing Statement

Shafan-Azhar. Psychological Therapy Outcomes and Engagement in People of Different Religions in England. *JAMA Netw Open*. Published April 08, 2025. doi:10.1001/jamanetworkopen.2025.4026

Data
Data available: No

Additional Information
Explanation for why data not available: This dataset contains personal National Health Service information and is not made routinely available.
